# The Gonococcal Genetic Island defines distinct sub-populations of *Neisseria gonorrhoeae*

**DOI:** 10.1099/mgen.0.000985

**Published:** 2023-05-22

**Authors:** Madison A. Youngblom, Abigail C. Shockey, Melanie M. Callaghan, Joseph P. Dillard, Caitlin S. Pepperell

**Affiliations:** ^1^​ Department of Medical Microbiology and Immunology, School of Medicine and Public Health, University of Wisconsin-Madison, Madison, WI, USA; ^2^​ Microbiology Doctoral Training Program, University of Wisconsin-Madison, Madison, WI, USA; ^3^​ Wisconsin State Laboratory of Hygiene, Madison, WI, USA; ^4^​ Department of Medicine (Infectious Diseases), School of Medicine and Public Health, University of Wisconsin-Madison, Madison, WI, USA

**Keywords:** *Neisseria gonorrhoeae*, Gonococcal Genetic Island (GGI), population genetics, pangenome

## Abstract

The incidence of gonorrhoea is increasing at an alarming pace, and therapeutic options continue to narrow as a result of worsening drug resistance. *Neisseria gonorrhoeae* is naturally competent, allowing the organism to adapt rapidly to selection pressures including antibiotics. A sub-population of *N. gonorrhoeae* carries the Gonococcal Genetic Island (GGI), which encodes a type IV secretion system (T4SS) that secretes chromosomal DNA. Previous research has shown that the GGI increases transformation efficiency *in vitro*, but the extent to which it contributes to horizontal gene transfer (HGT) during infection is unknown. Here we analysed genomic data from clinical isolates of *N. gonorrhoeae* to better characterize GGI+ and GGI− sub-populations and to delineate patterns of variation at the locus itself. We found the element segregating at an intermediate frequency (61%), and it appears to act as a mobile genetic element with examples of gain, loss, exchange and intra-locus recombination within our sample. We further found evidence suggesting that GGI+ and GGI− sub-populations preferentially inhabit distinct niches with different opportunities for HGT. Previously, GGI+ isolates were reported to be associated with more severe clinical infections, and our results suggest this could be related to metal-ion trafficking and biofilm formation. The co-segregation of GGI+ and GGI− isolates despite mobility of the element suggests that both niches inhabited by *N. gonorrhoeae* remain important to its overall persistence as has been demonstrated previously for cervical- and urethral-adapted sub-populations. These data emphasize the complex population structure of *N. gonorrhoeae* and its capacity to adapt to diverse niches.

## Data Summary

Isolates used for this study listed in TableS1. Raw sequencing data for these isolates can be obtained from NCBI. Protein sequences for MntH in *Neisseria gonorrhoeae* and *Neisseria meningitidis* can be found in the NCBI protein database under accessions WP_003698816.1 and WP_002229630.1, respectively.

**Impact Statement**

Cases of the sexually transmitted infection (STI) gonorrhoea are increasing rapidly, and widespread antibiotic resistance has left few viable treatment options. Spread of antibiotic resistance is probably facilitated by widespread horizontal gene transfer (HGT) among *Neisseria gonorroheae*. Here we identified differences between sub-populations of *N. gonorrhoeae* with and without the Gonococcal Genetic Island (GGI). The GGI has been shown previously to increase transformation efficiency and we found that bacteria encoding the element appear to have greater opportunities for HGT. We further found evidence of co-adaptation between *N. gonorrhoeae *’s core genome and the element. These results suggest that sub-populations with and without the element inhabit subtly different niches. Together, these findings show the importance of mobile genetic elements in shaping bacterial populations, and identify potential mechanisms used by *N. gonorrhoeae* to adapt to different host niches.

## Introduction

*Neisseria gonorrhoeae* is an obligate human pathogen and the causative agent of the sexually transmitted infection (STI) gonorrhoea. In 2019 the Centers for Disease Control (CDC) reported over 600,000 new infections with *N. gonorrhoeae,* with the rate of reported cases in the USA having risen by 92 % since a historical low in 2009 [1]. *N. gonorrhoeae* has been declared an urgent public health threat, as increasing rates of antibiotic resistance raise the spectre of untreatable forms of gonorrhoea.

*N. gonorrhoeae *’s propensity to engage in horizontal gene transfer (HGT) has facilitated the spread of antibiotic resistance. Transformation, the direct uptake and incorporation of exogenous DNA into the native chromosome, is the primary mechanism of HGT among *N. gonorrhoeae* [2]. There are two known mechanisms by which *N. gonorrhoeae* releases its DNA into the extracellular milieu: autolysis and via a Type IV secretion system (T4SS) [3]. *N. gonorrhoeae *’s T4SS secretes ssDNA across the cell envelope [3–5]. In an experimental mating system, a functional T4SS conferred 500-fold greater transformation efficiency than autolysis, indicating that secretion of DNA enables high rates of transformation in natural populations [5, 6].

Genes encoding the T4SS are located on a 59 kb genomic island called the Gonococcal Genetic Island (GGI), which is prevalent among *N. gonorrhoeae* (64–80 % of isolates were found to encode the element in prior surveys [7, 8]). The GGI, first described in *N. gonorrhoeae* reference strain MS11, contains 66 genes. Genes encoding the GGI T4SS share sequence similarity to the *Escherichia coli* F-plasmid T4SS, including 40 and 42% similarity between *traG* and *traH* genes, respectively [5]. Sequence characteristics of the GGI differentiate it from *N. gonorrhoeae *’s chromosome, including lower GC content, differing dinucleotide frequencies and a lower average number of DNA uptake sequences suggesting the GGI may have originally been acquired from a different species [3, 9]. Twenty-two genes in the GGI have been identified as essential to secretion and six other genes have sequence similarity to DNA binding and processing proteins, but the remaining 38 genes are non-essential for secretion and largely of unknown function [5, 6, 9].

Excision of the GGI is mediated by the site-specific recombinase XerCD [10]. XerCD recognizes the *dif* site, *difA*, found at one end of the GGI. A second, imperfect repeat of the sequence, *difB*, flanks the other side of the GGI. Loss, but not transfer, following excision of the GGI has been observed in experimental settings [3, 10]. Given that the GGI may have been laterally acquired and is capable of being excised from its host chromosome, it is possible the GGI was or is a mobile genetic element (MGE). Here we sought to characterize patterns of variation at the GGI locus, determine its mobility and investigate potential interactions with the host chromosome using whole genome sequence (WGS) data from natural populations of *N. gonorrhoeae *.

## Methods

### Data and genome assembly

Our goal for this study was an in-depth analysis of the GGI in a natural population. To accomplish this we sought to create a diverse dataset of *N. gonorrhoeae* genomes while also maintaining computational feasibility: our planned analyses included delineating gene content of a genomic island and ancestral reconstruction, both of which are unmanageable with large sample sizes. To accomplish this we downloaded a phylogenetic tree associated with a recent *N. gonorrhoeae* dataset from Pathogenwatch (https://pathogen.watch/collection/6s7iszmj1edv-mortimer-et-al-2020). Using Treemmer v 0.3 [11] we subsampled the tree to 200 isolates (from 860 in total) while maintaining maximum genetic diversity. Using the NCBI accessions from our subsampled tree we downloaded raw sequencing data from NCBI. The quality of the raw sequencing data was verified using FastQC v 11.8.0 [12]. We *de novo* assembled the isolates using SPAdes v 3.13.1 [13], contigs with <5× coverage and/or <500 bp were removed and assembly quality was assessed using Quast v 5.0.2 [14] to ensure all assemblies had an N50 >50 kb. Genomes were annotated with Prokka v 1.14.0 [15]. We downloaded the complete genome for strain NCCP11945 (GenBank accession: CP001050.1) and added it to our sample so we could compare any variants to a completed genome, resulting in a total of *n*=201 (Table S1).

### Pan-genome calculations and core genome tree

We used Roary v 3.12.0 [16] with standard parameters to perform a pangenome calculation on all of the annotated genomes, sorting genes by frequency into core (≥99 % frequency), soft core (95 % ≤ frequency < 99 %), shell (15 % ≤ frequency < 95 %) and cloud genomes (frequency <15 %); these are the default parameters for pangenome calculation. Additionally, this analysis resulted in the total core genome alignment. Additional pangenome calculations were performed with GGI+ and GGI− isolates separately (GGI+ and GGI− core genome alignments, respectively). Core genome alignments from the total sample, GGI+ and GGI− samples were used to infer phylogenetic trees using RAxML v 8.2.3 [17] with the General Time Reversible (GTR) model of nucleotide substitution with the CAT approximation of Gamma model of rate heterogeneity. We also used the results of the total pangenome analysis to characterize differences in accessory gene content between GGI+ and GGI− isolates. Using the separate GGI+ and GGI− gene presence/absence data from Roary we created rarefaction (core genes) and accumulation (pan genes) curves in R using published scripts [18] to randomly subsample the larger GGI+ dataset to the size of the GGI− dataset (*n*=78), 100 times without replacement and plotting the average number of core and pan genes as a function of the number of isolates. Additionally, we used these data to make a heatmap showing accessory gene frequencies in both groups. SNP alignments were made for the separate core genome alignments from GGI+ and GGI− isolates using SnpSites v 2.4.1 [19] and phylogenetic networks of the alignments were made using SplitsTree v 4.14.5 [20].

### Determining GGI presence/absence

We used blast to determine presence or absence of the GGI in our isolates. We used the nucleotide sequences of GGI genes as annotated in *N. gonorrhoeae* strain MS11 (66 genes in total, starting with *yaa* and ending with *parA*) to identify GGI genes in our dataset. Using Blast v 2.9.0 [21] we were able to clearly identify GGI genes as they do not exhibit homology with any *N. gonorrhoeae* genes outside the element. We filtered results to include only alignments ≥50 % of the full length of the gene. Using this method, we identified that 123/201 (61 %) of our isolates are GGI+, maintaining anywhere from 88 to 100 % of GGI genes per isolate. The GGI gene map was plotted using genoPlotR [22].

### Recombination analyses

We used SplitsTree to visualize core genome phylogenetic networks and to perform the phi-test for recombination [20]. We used Gubbins v 2.3.2 [23] to identify recombination breakpoints in core genome alignments and visualized these breakpoints using Phandango [24]. We calculated the proportion of the core genome alignment affected by recombination, as well as the proportion of recombinant sites per genome, using custom python scripts (https://github.com/tatumdmortimer/recombination). Branch lengths were calculated from core genome phylogenies made wtih RAxML using ggtree [25].

### Genome-wide association study: *F*_ST_ outliers and accessory gene association

We calculated Weir and Cockerham’s *F*_ST_ for bi-allelic SNPs in the core genomes of GGI+ and GGI− populations using vcflib (https://github.com/vcflib/vcflib). We permuted this analysis 100 times and used the maximum *F*_ST_ observed in the null distribution as a cut-off to identify *F*_ST_ outliers. We used Scoary v 1.6.16 to calculate the strength of association between accessory gene content and the GGI as a trait [26]. We used the Bonferroni correction to account for multiple comparisons (*P*-value cutoff of <0.05) and performed 100 permutations of the analysis. Top Scoary results were annotated using a blast search of protein sequence and pulling functions from the top hits. Data and scripts for the *F*_ST_ outlier analysis and plot can be found at https://github.com/myoungblom/Evolution_of_the_GGI.git.

### Identification of homoplasies

We used TreeTime v 0.9.4 to perform ancestral reconstruction of SNPs on the core genome phylogeny and identify homoplastic SNPs (SNPs that arose more than twice on the phylogeny) [27]. Data and scripts for calculation of homoplastic SNPs can be found at https://github.com/myoungblom/Evolution_of_the_GGI.git.

### Analysis of mobile genetic elements

We used ProphET [28] to detect prophages in our collection of genomes. Briefly, ProphET performs a similarity search of annotated proteins from bacterial genomes against a database of known phage proteins to identify prophages within bacterial genomes. ProphET discards regions with a low density of phage-associated genes. After identifying the prophage regions, we calculated pairwise mash [29] distances, which are based on shared k-mer (sequences of length k) content between prophage nucleotide sequences. To visualize the relatedness between prophage regions in GGI+ and GGI− isolates we performed a multi-dimensional scaling analysis of calculated mash distances. MobileElementFinder v 1.0.3 was used to annotate MGE and all ‘putative’ elements were removed before analysis, leaving only ‘predicted’ insertion sequences [30]. Data and scripts for phage and MGE analysis can be found at https://github.com/myoungblom/Evolution_of_the_GGI.git.

### Core GGI alignment

To create a core GGI gene alignment we identified genes within the GGI present in ≥99 % of GGI+ isolates for a total of 53 genes. Multiple-sequence alignments were made for each gene using Prank v 0.150803 [31]. Then, in the order specified by the MS11 GGI, core GGI gene alignments were concatenated for each isolate into the core GGI alignment. A phylogenetic tree of this alignment was inferred using RAxML v 8.2.3 [17] with the GTR model of nucleotide substitution with the CAT approximation of Gamma model of rate heterogeneity. Phylogenetic trees were plotted using ggtree [25]. A SNP alignment of the core GGI alignment was made using SnpSites [19] and a phylogenetic network was created and plotted using SplitsTree [20].

### Calculating diversity statistics

Diversity statistics including nucleotide diversity ( π ), theta estimator ( θ ), Tajima’s *D* (*D*) and π N/ π S were calculated using Egglib v 3.0.0 [32]. Data and scripts for calculating π N/ π S for genes in the GGI can be found at https://github.com/myoungblom/Evolution_of_the_GGI.git

### Ancestral reconstruction of the GGI

We used adegenet v 2.1.3 [33] to perform principal component analysis (PCA) on the GGI’s core genes (present in ≥99 % of GGI+ isolates). We reconstructed gains and losses of the GGI on the maximum-likelihood phylogeny using ape v 5.4 [34]. We defined gains and losses as a change in the majority (>50 %) of reconstructed ancestral states from parent to child node. Data and scripts for ancestral reconstruction can be found at https://github.com/myoungblom/Evolution_of_the_GGI.git.

## Results

### Identification of the GGI among isolates of *N. gonorrhoeae*

**Fig. 1.**
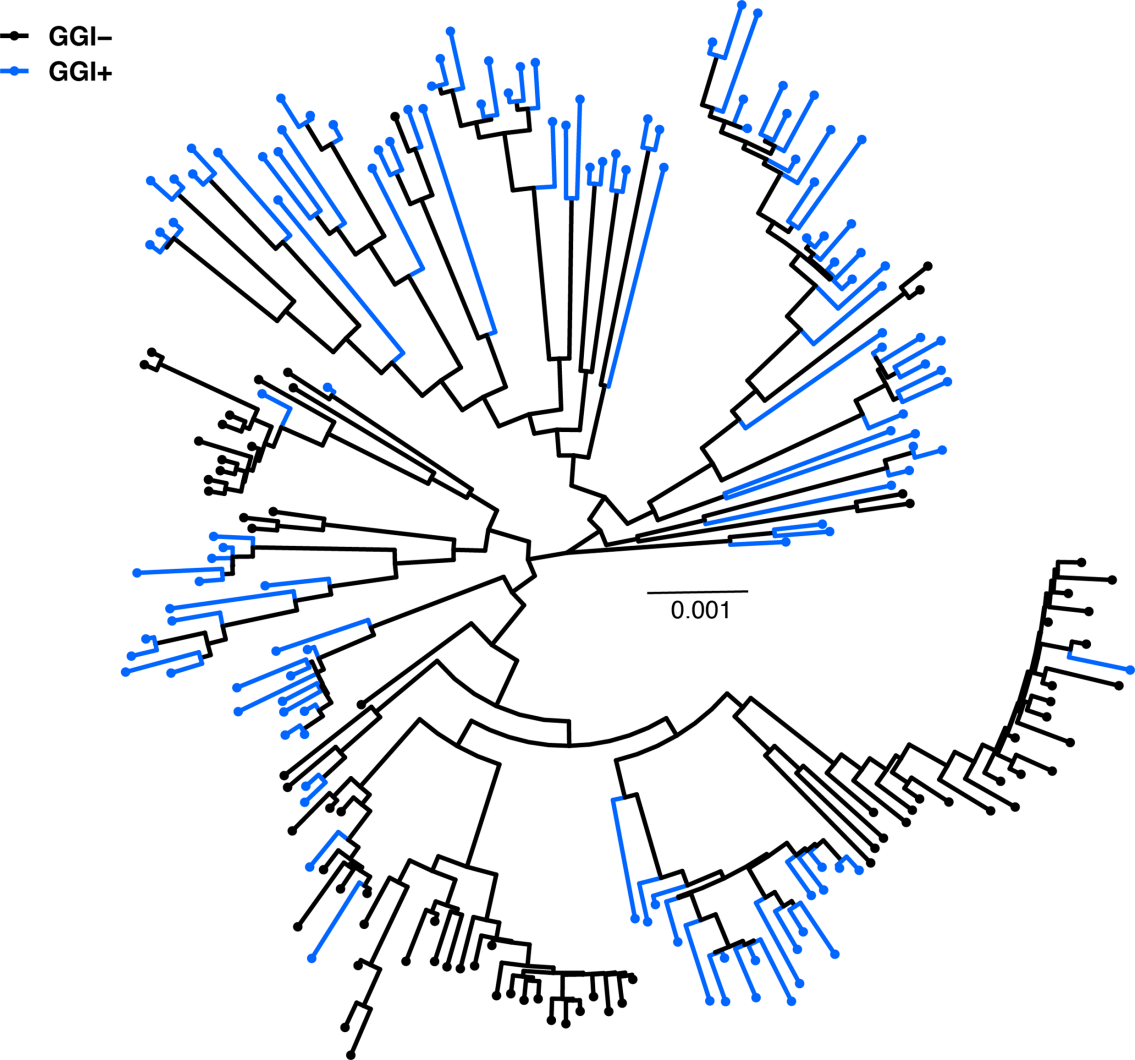
The core genomes of *N. gonorrhoeae* GGI+ and GGI− isolates are genetically differentiated. Core genome phylogeny inferred using RAxML; tips are coloured by presence or absence of the GGI. In total, there were 201 isolates (including reference strain NCCP11945) with 123 being GGI+ and 78 being GGI−. Tree is midpoint rooted and scale bar represents nucleotide substitutions per site.

Our goal for this study was to perform an in-depth analysis of the diversity and mobility of the GGI in a natural population. For this, we needed a relatively small sample size to maintain computational feasibility. To achieve this we sampled from a large dataset of *N. gonorrhoeae* isolates collected in New York City as a part of a general public health surveillance project [35]. New York City is a large and cosmopolitan city, and using this sample allowed us to gather a relatively diverse collection of genomes while maintaining uniformity with respect to recruitment, sampling, associated metadata, sequencing methodology and quality. We informatively subsampled the full dataset of 860 isolates to retain diversity in our subsample of 200 (Fig. S1; Table S1, available in the online version of this article). After *de novo* assembly and annotation, we performed a pangenome analysis that identified a pangenome size of 3205, with 1742 core genes (Table S2), which is in agreement with other recently published studies [36, 37]. This indicates that ~15 % of the gene content within each genome is variable, and accessory genome content appears to be skewed towards rare frequencies, with over 1000 ‘cloud’ genes (defined as a frequency <15 % in the sample) (Table S2). We used an alignment of core genes from our sample to infer a phylogeny (Fig. 1).

We used two methods to identify the GGI in our sample: pangenome analysis and blast using nucleotide sequences of the GGI from *N. gonorrhoeae* laboratory reference strain MS11 (comprising 66 genes). We found that 123 (61 %) out of 201 isolates in our sample were GGI+. This is lower than a prior estimate based on PCR identification of the genes *atlA* and *traG,* that found 64–80 % of isolates encoded the island [5]. The strain collection used in the aforementioned study by Dillard and Seifert was chosen to include a significant number of isolates associated with severe infection (e.g. pelvic inflammatory disease and disseminated gonococcal infection), and their findings showed an association between presence of the GGI and disease severity, which could explain why their estimate of GGI prevalence was higher than ours [5]. Mapping GGI presence onto the core genome phylogeny shows the phenotype is associated with clusters, with some intermingling of GGI+ and GGI− within sub-clades (Fig. 1).

### GGI+/− pangenomes and diversity

**Fig. 2.**
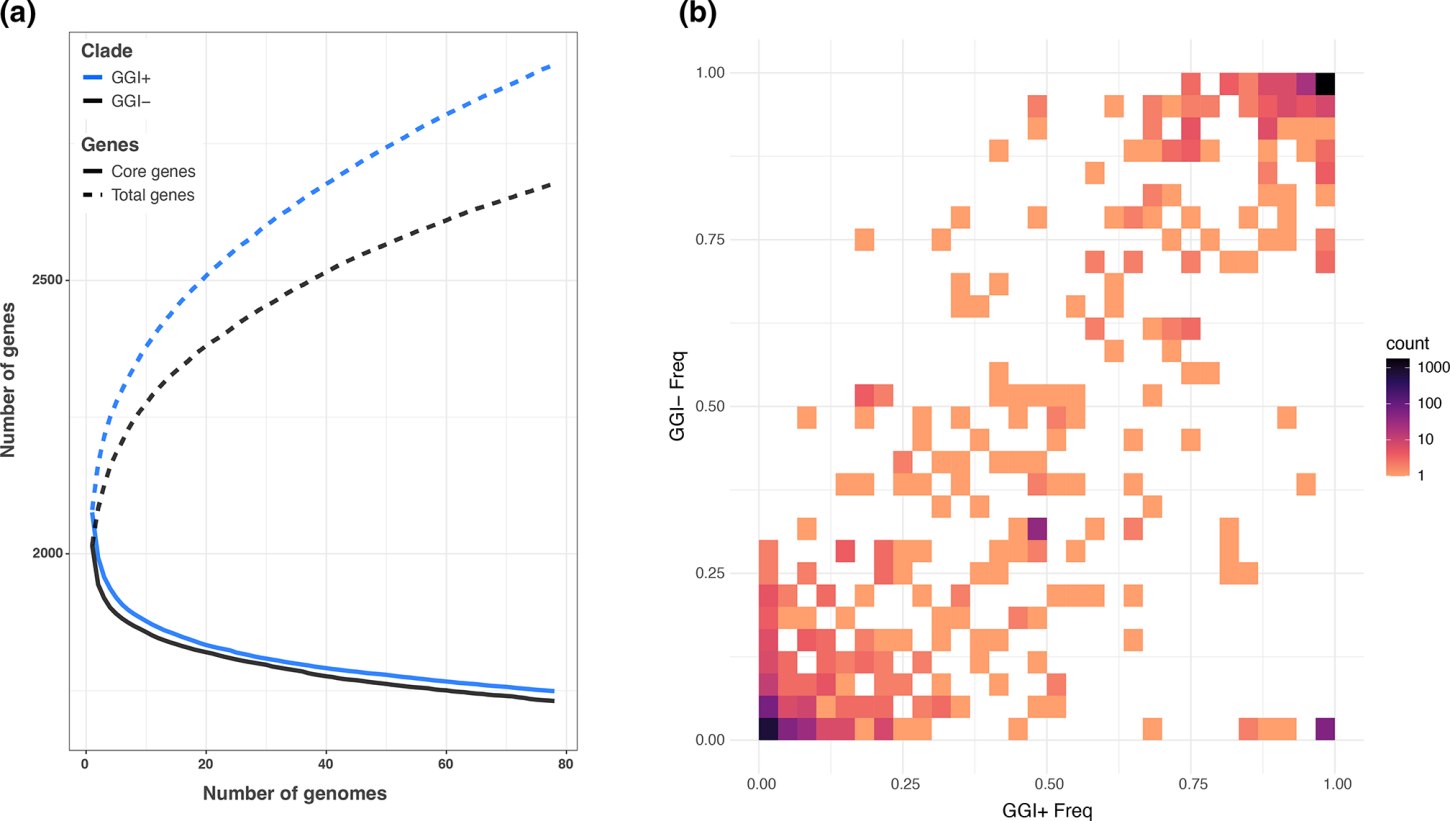
The pangenome of GGI+ isolates is larger than GGI− isolates while accessory gene content is shared between groups. (**a**) Rarefaction and accumulation curves of core and total gene content in GGI+ and GGI− isolates show a larger pangenome among GGI+ isolates. All groups contain an equal number of isolates: GGI+ isolates were randomly sampled without replacement 100 times to the size of the GGI− dataset (*n*=78). (**b**) Heatmap comparing accessory gene frequencies in GGI+ and GGI− groups. Accessory genes are maintained at similar frequencies between the two populations (except for the GGI, shown in purple in the bottom right of the plot), as indicated by the density of genes along the diagonal (equal frequencies in both populations). This suggests that there are no barriers to accessory gene flow between groups.

To identify genomic features associated with the presence of the GGI we first performed pangenome analyses on GGI+ and GGI− isolates and found that the average number of genes per isolate as well as the number of core genes is similar between subgroups, with GGI+ isolates encoding an average of 77 more genes than GGI− isolates (a difference largely made up of the GGI itself) (Table S2). We identified a larger pangenome in the GGI+ group, indicating a more diverse pool of accessory genes (Fig. 2a; Table S2); however, accessory genes that are shared between them (~1500 genes) are maintained at similar frequencies, suggesting there are no barriers to accessory gene flow between groups (Fig. 2b). Additionally, measures of genetic diversity within and between the two groups are similar, providing further support for the hypothesis that accessory genes are shared freely between GGI+ and GGI− isolates (Fig. S2).

**Fig. 3.**
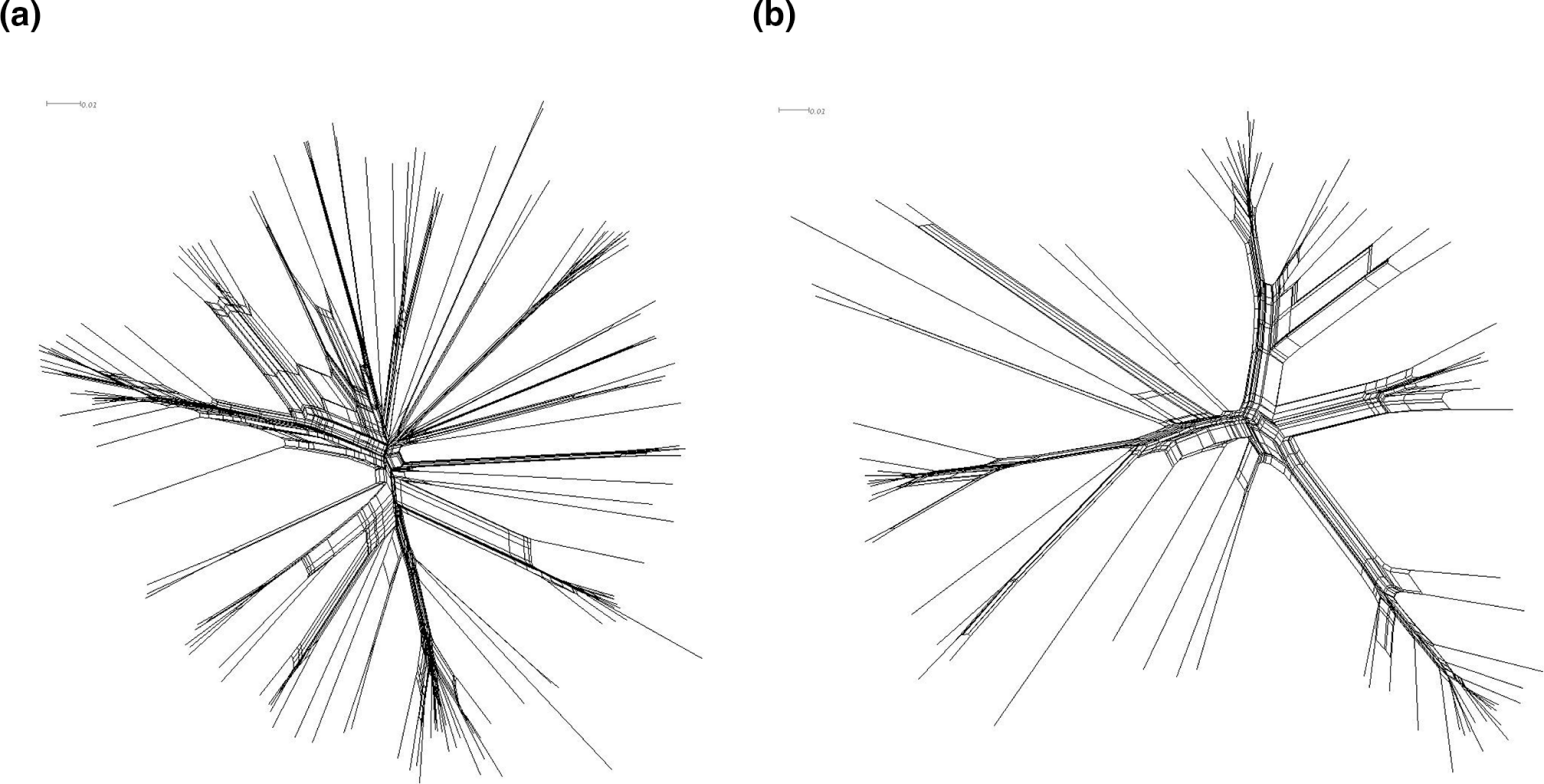
Core genome networks indicating differences in patterns of recombination between GGI+ and GGI− isolates. Core genome networks from GGI+ (**a**) and GGI− (**b**) isolates made using SplitsTree. The PHI test for recombination was significant (*P*<0.05) for both groups, indicating evidence of recombination. Network scale bars represent substitutions per site.

### Recombination in the core genomes of GGI+ and GGI− isolates

Secretion of ssDNA by the GGI has been hypothesized to aid in natural transformation of *N. gonorrhoeae *. To investigate a potential impact of the GGI on recombination, we inferred and characterized patterns of homologous recombination within our sample. We used Gubbins [23] to identify recombinant fragments in the core genome alignment of our complete sample and found over 3000 fragments widely dispersed throughout the core genome of *N. gonorrhoeae* (Fig. S3). Several recombinant fragments are shared by the entire sample, suggesting inheritance from a common ancestor, while most fragments are shared by only a few isolates, suggesting a more recent transfer (Fig. S3). Results from this analysis indicate that 81 % of sites in the core alignment are predicted to be within a recombinant fragment in at least one isolate, supporting previous observations of frequent recombination among *N. gonorrhoeae* [38]. Next, we investigated whether patterns of recombination differed between GGI+ and GGI− isolates through analysis of their respective core genome alignments. The pairwise homoplasy index (PHI) test for recombination was significant (*P*<0.05) for both groups, indicating the presence of recombination in both sub-populations. We observed the phylogenetic networks of core genomes for GGI+ and GGI− isolates as having slightly different structures: the GGI+ network has a more ‘star-like’ shape (Fig. 3a) than the GGI− network (Fig. 3b). Star-like shapes occur with rapid accumulation of mutations, consistent with relatively rapid expansion of the GGI+ sub-population.

**Fig. 4.**
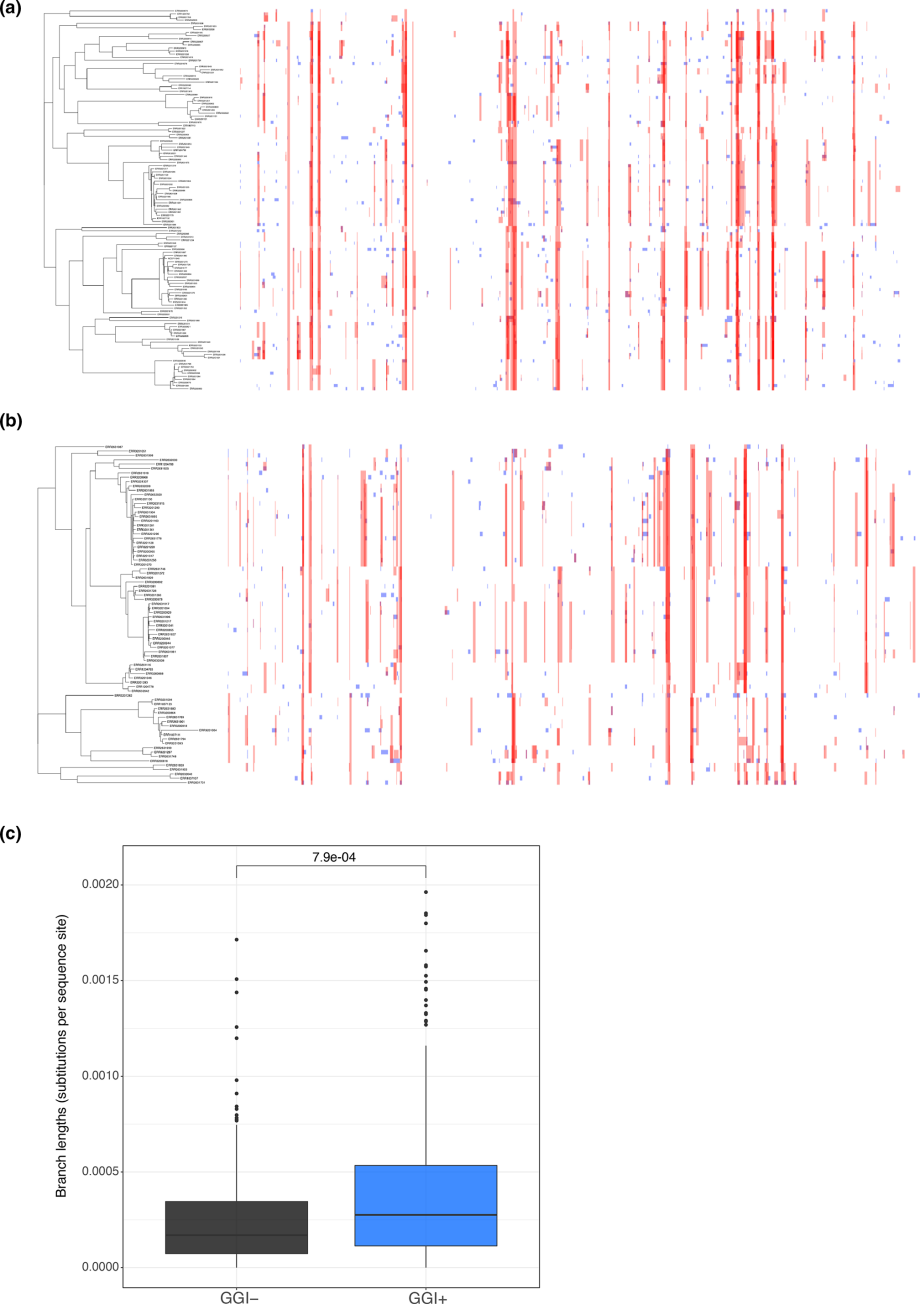
Isolates have similar frequencies of recombination within each group, but GGI+ isolates appear more differentiated from one another. Recombination tracts predicted by Gubbins in the separate core genomes of GGI+ (**a**) and GGI− (**b**) isolates, plotted with a recombination-adjusted tree using Phandango. In total, 59 % of the GGI− core alignment and 68 % of the GGI+ core alignment was affected by recombination. Recombinant fragments shared between isolates within the dataset are shown in red, while those which are determined to have originated outside of the dataset are in blue. Branch lengths in the core genome phylogeny of GGI+ isolates are significantly longer than GGI− isolates (Mann–Whitney U test, *P*=7.9e-04) (**c**). These data indicate different evolutionary histories between the two groups, and greater core genome differentiation among GGI+ isolates.

To examine differences in recombination more thoroughly, we annotated recombinant fragments in each of the GGI+ and GGI− core genomes separately and found a modest difference in the proportion of the alignment affected by recombination: 68 and 59 % for GGI+ and GGI−, respectively. The tendency for a particular recombinant fragment to be shared across isolates does not differ between GGI+ and GGI− isolates (Fig. S4), but there does appear to be a modest but significant difference in recombinant fragment length, with GGI+ isolates having larger fragments (median length of 1653 for GGI+ compared to 1225 for GGI−; Mann–Whitney U test *P*=1.2e-05; Fig. S5). Together, these data indicate that there are subtle differences in recombination between GGI+ and GGI− isolates. We noted that the branches of GGI+ isolates appeared longer on average (Fig. 4a) than the branches on the GGI− tree (Fig. 4b) in recombination-adjusted phylogenies. We used ggtree [25] to calculate total branch lengths of each tree and found that branches of the GGI+ core genome phylogeny were significantly longer than those of the GGI− phylogeny (Fig. 4c). The core genome variants in GGI+ isolates are skewed toward rare frequencies, reflected in more negative values of Tajima’s *D* and a higher value of Watterson’s estimator ( θ ) for the GGI+ core alignment (Table S2). Similar to patterns of variation in the accessory genome, this is consistent with GGI+ isolates accessing a more diverse pool of partners for HGT, enabling importation of novel fragments into the core genome.

### Associations between the GGI and variants in the core and accessory genomes of *N. gonorrhoeae*

**Fig. 5.**
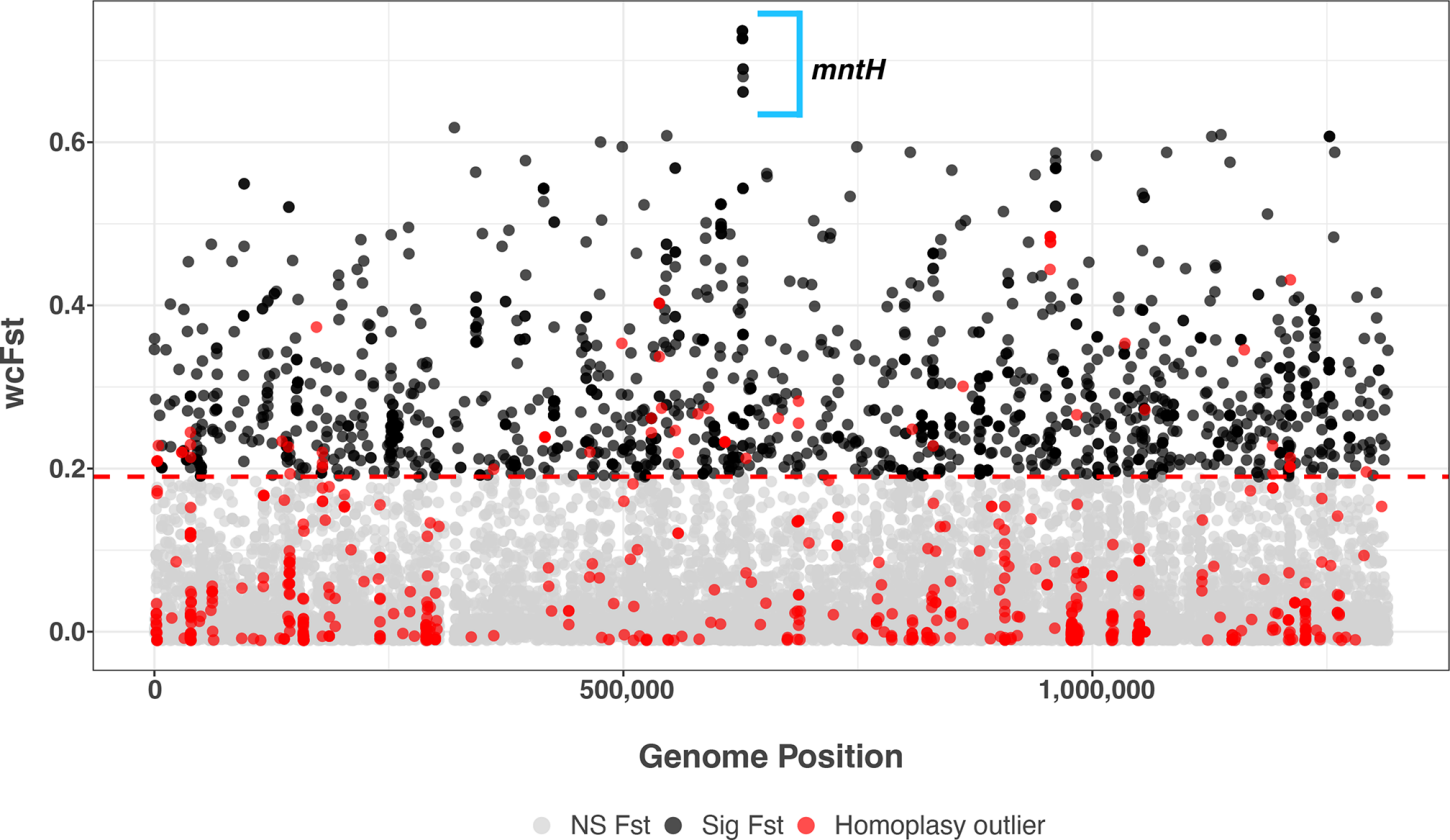
Multiple variants in *mntH* associated with GGI presence. Weir and Cockerham's *F*_ST_ (wcFst) values of core genome variants are plotted against the core genome position. The significance threshold was estimated by taking the maximum wcFst value from 100 random permutations of phenotypes. Variants with non-significant wcFst values are shown in grey, and those with significant values in black. Additionally, variants that are homoplasy outliers (see Methods) are shown in red. Twelve variants all within *mntH* have markedly high wcFst values, indicating a strong association with the presence of the GGI.

Genetic differentiation of GGI+ and GGI− sub-populations could reflect infrequent gain/loss of the element (e.g. because of mechanistic constraints on mobility). Another, not mutually exclusive, explanation is that there is co-adaptation of the *N. gonorrhoeae* genome to the element. To investigate the latter hypothesis, we performed a genome-wide association study (GWAS) to query both the core and accessory genomes for variants associated with the GGI. To identify core genome variants associated with presence of the GGI, we performed an *F*_ST_ outlier analysis. We identified 1998 variants (out of a total of 22 839 total core genome variants) with significant differences in allele frequency between the GGI+ and GGI− sub-populations (Fig. 5). Notably, a cluster of 12 variants are outliers in the distribution of *F*_ST_ values and all 12 lie within the same gene (Fig. 5). This gene was initially annotated as a hypothetical protein. Results of a blastp search revealed the presence of a domain from the family of natural resistance-associated macrophage proteins (Nramps), which function as divalent metal ion transporters commonly involved in manganese and iron homeostasis [39, 40] and are found ubiquitously across the eukaryotic and bacterial domains. In addition to being conserved across strains of *N. gonorrhoeae* (NCBI protein accession WP_003698816.1) this gene is also present in *N. meningitidis* (NCBI protein accession: WP_002229630.1). In other bacterial species this gene is known as *mntH*, which is the notation we will use in this study. To our knowledge this is the first description of *mntH* in the *N. gonorrhoeae* genome, as a previous bioinformatic search prior to the advent of widespread WGS yielded no results [41]. Isolates with all 12 of the SNPs identified as *F*_ST_ outliers produce a version of MntH containing 11 amino acid changes spread across the entire length of the protein (Fig. S6). The variants of *mntH* frequently co-occur in a given isolate (i.e. isolates having variants in *mntH* usually have multiple variants), suggesting that they act interdependently, and demonstrate a strong association with GGI presence/absence when mapped to a core genome phylogeny (Fig. S8).

To investigate whether there was evidence of positive selection on the *F*_ST_ outliers we sought to identify homoplastic mutations within the core genome. Homoplastic variants arise independently more than once on the phylogeny; a potential cause is positive selection, whereby advantageous mutations occur repeatedly [42]. Using ancestral sequence reconstruction with TreeTime [27] we identified 16 753 core genome variants as homoplastic (i.e. having arisen two or more times on the phylogeny). The high proportion of homoplastic mutations probably reflects frequent HGT in *N. gonorrhoeae *. We used the distribution of values of mutation multiplicity to define variants with extreme values (defined here with a 95^th^ percentile cutoff) as putative candidates under positive selection (distribution of mutation multiplicity shown in Fig. S7). We did identify some overlaps between variants with significant differentiation between GGI+ and GGI− sub-populations and those with significant homoplasy counts (Fig. 5), but this overlap appeared random and did not include variants at the highest end of the *F*_ST_ distribution. We conclude that these analyses do not provide evidence of positive selection on GGI-associated variants, at least as measured with homoplasy counts.

Given the substantial variation in accessory gene content we observed among isolates of *N. gonorrhoeae *, as well as the association of core genome variants with the presence of the GGI, we hypothesized that the accessory genome could also show signs of interaction with the element. We used Scoary [26] to calculate the strength of association between genes in the accessory genome and presence/absence of the GGI. We identified 10 genes that were strongly associated with GGI presence/absence (Table S3). Annotation of the genes via blast search revealed two of these genes encode phage proteins: recombination protein *ninB* and a phage tail protein (phage P2, protein I), both of which are ubiquitous among Gram-negative bacteria. We used ProphET [28] to annotate prophages in the genomes of our sample and found that neither of these genes was uniformly associated with an intact prophage genome.

### Associations between mobile elements and the GGI

**Fig. 6.**
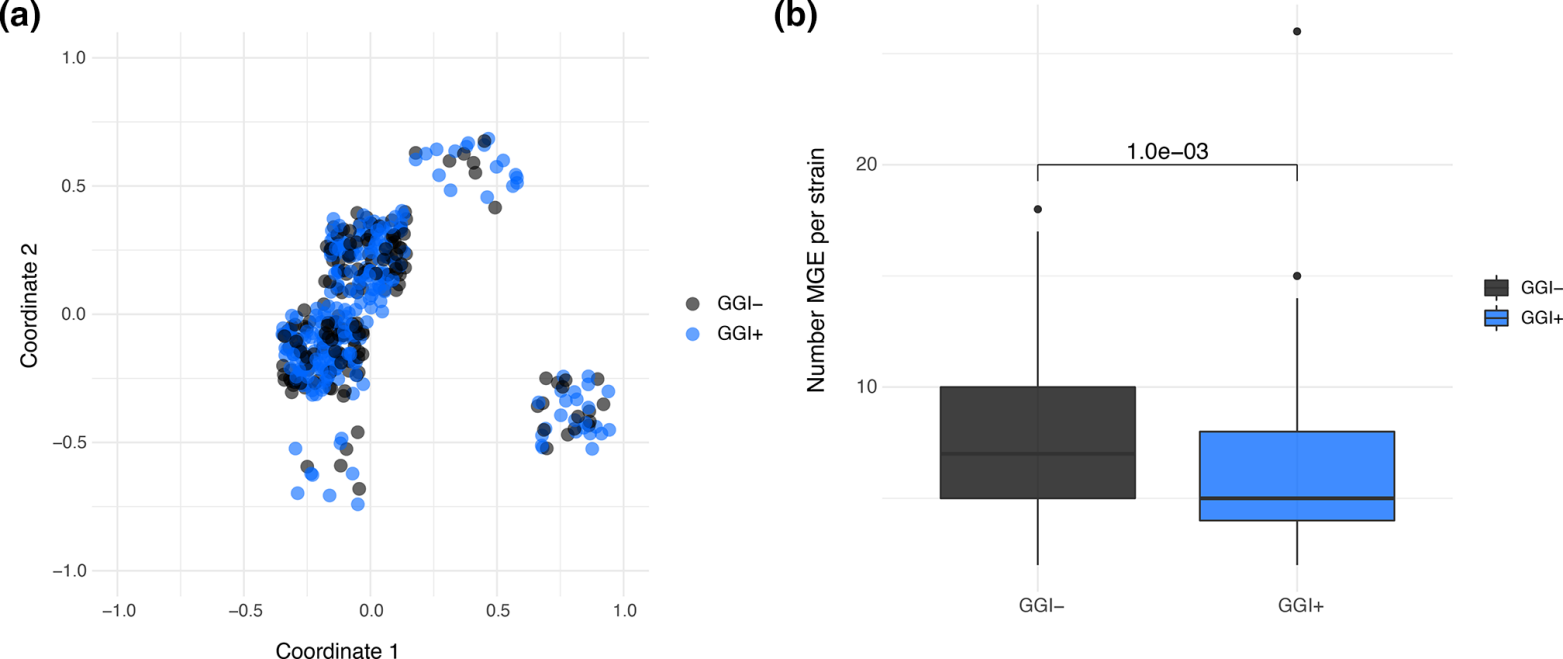
Mobile genetic elements (MGEs) accumulate in GGI− isolates while prophages are exchanged freely. (**a**) Multi-dimensional scaling (MDS) of pairwise mash distances of prophage sequences as annotated by ProphET. Each point represents a single annotated prophage from a given isolate, coloured by GGI presence in that isolate. Prophage clusters are not separated by GGI presence/absence, indicating that prophages are exchanged between groups. (**b**) MGEs (specifically insertion sequences; ISs) were annotated using MobileElementFinder and the total number of ISs per strain was determined and plotted by GGI presence/absence. A Mann–Whitney U test shows significantly higher numbers of MGEs per strain in GGI− isolates (*P*=6.6e-04).

Given the association we observed between the GGI and phage proteins, we hypothesized that phage or other mobile elements could differentiate GGI+/− sub-populations. To test this hypothesis, we calculated pairwise mash distances between prophage sequences annotated by ProphET and then performed multi-dimensional scaling (MDS) to look at the relatedness of phage sequences between GGI+ and GGI− isolates (Fig. 6a). MDS revealed approximately five clusters of prophages, which probably correspond to previously identified dsDNA prophages Ngo Φ 1–5 [43]. Despite the association of two phage genes with the GGI, we do not identify an association between the GGI and any of the phage clusters, suggesting that prophages move readily between GGI+/GGI− sub-populations. We used MobileElementFinder [30] to annotate insertion sequences (ISs) in our sample and investigate whether these elements are associated with the GGI. We found GGI− isolates to have significantly more MGEs per strain than GGI+ isolates (Fig. 6b), suggesting that bacteria lacking the element are permissive of IS invasion and proliferation.

### Diversity of the GGI

**Fig. 7.**
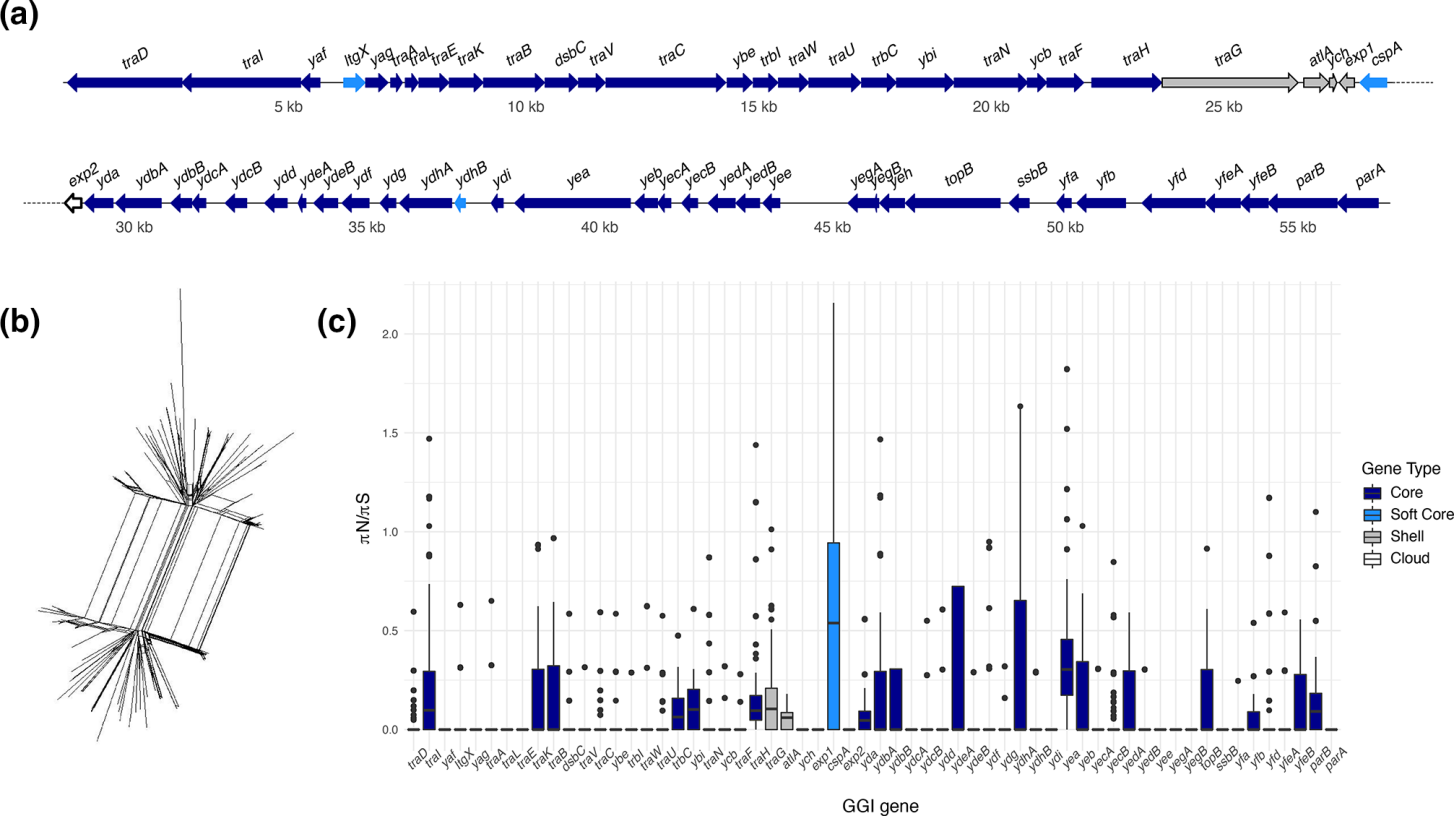
The GGI is largely conserved with minimal variation in gene content and sequence. (**a**) Locus map showing the pangenome of the GGI is skewed towards core genes, with only a few accessory GGI genes. Dark blue genes are core GGI genes (≥99 % frequency), light blue are soft core (95–99 % frequency), grey are shell (15–95 % frequency) and white are cloud (<15 % frequency) accessory GGI genes. Dotted line indicates the continuation of the operon on the next line. (**b**) Core GGI network revealing evidence of intra-locus recombination. Network of core GGI genes (53 in total) from GGI+ isolates made in SplitsTree. The PHI test for recombination was significant (*P*<0.05) indicating evidence of recombination. (**c**) Pairwise π N/ π S values for GGI genes indicating varying selective pressures between genes. Nucleotide diversity values at non-synonymous and synonymous sites calculated with Egglib for each pairwise comparison of the same GGI gene between two GGI+ isolates. Values are plotted as boxplots with outliers shown as points.

In our dataset we identified an average of 60 GGI genes per GGI locus, with 53 of those genes defining a core GGI (i.e. present in >99 % of GGI+ isolates) (Fig. 7a; Table S2). This suggests that only a handful of GGI genes are dispensable, notably the region spanning *traG* to *exp1* (coloured in grey, Fig. 7a). These results are consistent with previous studies that characterized gene content conservation in the GGI, both in *N. gonorrhoeae* [5] and in *N. meningitidis* [44]. Interestingly, two genes known to be essential for DNA secretion, *traG* and *atlA* [6, 45–47], while found at high frequencies of 93 and 85% respectively, are not within the core genome of the element. This suggests that maintaining the GGI has benefits beyond its capacity to secrete DNA into the surrounding environment and that sub-populations of GGI+ isolates with limited capacity for DNA secretion can persist in *N. gonorrhoeae *’s natural niche.

To further investigate diversity of the GGI we inferred a phylogenetic network from an alignment of concatenated core GGI genes (Fig. 7a). The network exhibits significant reticulation, indicating intra-locus recombination (Fig. 7b). Comparison of gene by gene values of π N/ π S reveals a modest amount of variation among genes of the GGI, with the preponderance evolving under purifying selection. Patterns of variation in GGI accessory genes were similar to core genes (Fig. 7c).

### Ancestral reconstruction of the GGI

**Fig. 8.**
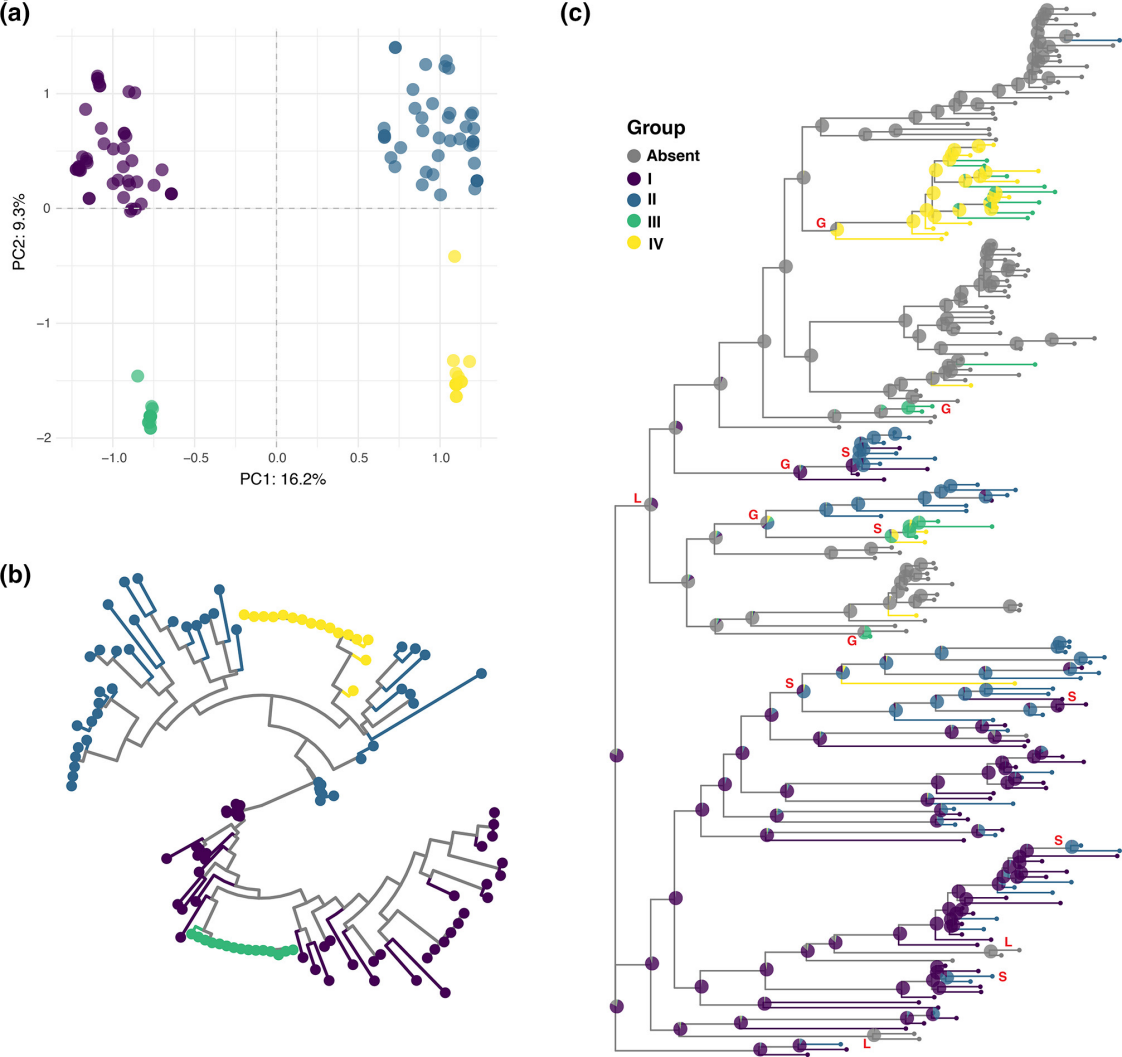
GGI is gained and lost, and switched between four different subtypes. (**a**) PCA of core genome alignment of 53 genes present in ≥99 % of GGI+ isolates. Analysis was performed using the adegenet package in R showing that the GGI clusters into four distinct groups (named I–IV). (**b**) Maximum-likelihood phylogeny inferred using RAxML of core GGI gene alignment, coloured by GGI group. (**c**) Ancestral state of the GGI locus was inferred using the R package Ape, plotted as a phylogenetic tree inferred using RAxML. Ancestral state probabilities are labelled at each node using a pie chart, coloured according to GGI group. The state of the GGI changes a total of 14 times on this phylogeny which are marked by red letters: G (gained) five times, L (lost) three times and S (switched) GGI group six times.

Presence of the GGI is structured on the core genome phylogeny (Fig. 1), with GGI+ and GGI− isolates forming clusters and we wondered whether this structure would extend to distinct types of GGI. PCA of genetic diversity within the core genome of the GGI delineated four distinct groups, which we refer to as I–IV (Fig. 8a); the corresponding phylogeny of the GGI core genome is shown in Fig. 8b.

We performed an ancestral reconstruction of GGI status using Ape [34] to estimate the number of gains, losses and exchanges of the GGI during evolution of our sample (Fig. 8c). Using this approach, we identified five gains, three losses and six ‘switch’ events during diversification of our sample of 201 isolates (Fig. 8c). We believe these switch events indicate that part or all of the GGI may be switched out for another type, without requiring the loss of the entire element, and subsequent gain of another type of GGI.

## Discussion

The GGI is a~59 kb genetic element variably present in *N. gonorrhoeae* [5] and in some other species of *Neisseria* [44]. The GGI as first described in *N. gonorrhoeae* MS11 contains 66 genes, some of which encode a T4SS that secretes ssDNA [5, 45]. *In vitro,* a functioning T4SS conferred 500-fold higher transformation efficiency than autolysis [5, 8], which led to the hypothesis that the GGI augments homologous recombination among natural populations of *N. gonorrhoeae* by increasing the amount of DNA available for transformation in the environment.

Recent work identified increased expression of the GGI in adherent cells [48], indicating that DNA secretion may be upregulated in biofilms. Additionally, we know that DNA secreted by the T4SS is involved in the initial stages of biofilm development [49] and is an important component for the stability of biofilms. Of the 66 genes encoded by the GGI, 22 have been shown to be essential for T4SS function, and the remaining 38 genes are of unknown function [6, 9].

Here we analysed genomic data from a natural population of *N. gonorrhoeae* in New York City [35] and found the GGI segregating at intermediate (61%) frequency, with an ‘all or nothing’ pattern with respect to gene content within the element (Figs 1 and 7a; Table S2). Presence/absence of the GGI is structured on the core genome phylogeny, indicating that isolates with and without the element form genetically differentiated groups (Fig. 1). These sub-populations do not, however, appear to be genetically isolated, as we found evidence that accessory gene content, including phage genes, are exchanged between groups (Figs 2b, 6a and S2).

Our results indicate that while inter-genomic recombination is common across our sample of *N. gonorrhoeae *, bacteria lacking the GGI appear to have fewer opportunities to acquire novel variants via HGT. The evidence supporting this hypothesis comes from our observations that the GGI+ sub-population has a larger pangenome (Fig. 2), and higher diversity of its core genome relative to GGI− isolates (Fig. 4). Core genome variants in the GGI+ sub-population also exhibit a stronger skew toward rare frequencies as shown by lower values of Tajima’s *D* (Table S2). Greater diversity of accessory gene content and core genome variants suggests isolates encoding the GGI have access to a larger and/or more diverse pool of partners for HGT. Finally, we found evidence that GGI− isolates accumulate more IS elements than isolates encoding the element (Fig. 6b). This latter phenomenon is typical of bacteria undergoing host adaptation and consequent niche restriction [50–54]. We identified similar patterns of diversity in our comparative genomic study of host adaptation among *Mycobacterium abscessus *, where host adapted/niche-restricted strains appeared to have fewer opportunities to engage in HGT than environmental *M. abscessus* [55]. *N. gonorrhoeae* differs from *M. abscessus* in that it is a strict human pathogen, and we posit that observed differences between GGI+ and GGI− sub-populations reflect differential access to niches within the human body containing diverse pools of bacteria.

### Core genome co-adaptation

We hypothesized that the adaptive forces separating GGI+ and GGI− isolates could have affected the *N. gonorrhoeae* genome beyond the element itself. To investigate this, we first performed *F*_ST_ outlier analysis to identify candidate variants in the core genome mediating compensation for the element and/or adaptation to distinct niches occupied by the two sub-populations (Fig. 5). The results of this analysis are striking: variants at the most extreme tail of *F*_ST_, clear outliers from the distribution, comprise 12 SNPs within a single *N. gonorrhoeae* core gene that we have termed *mntH* (Fig. 5). MntH belongs to the family of Nramp proteins, which function as divalent metal ion transporters. To our knowledge this is the first identification of an *mntH* orthologue in *N. gonorrhoeae* [41]. The 12 SNPs within *mntH*, which result in 11 non-synonymous amino acid substitutions spread over the 417 aa protein, were largely found in an ‘all or nothing’ pattern similar to the GGI itself within a given isolate (Figs S6 and S8). This suggests that the variants interact with each other with potential impacts on protein function given the large number of coding variants.

Nramp proteins are an essential part of iron (Fe) and manganese (Mn) metabolism in many species [40]. These metals are essential enzyme cofactors, particularly for the function of proteins protective against reactive oxygen species [41, 56, 57]. Nramp proteins play an important role at the host–pathogen interface [58] where there is strong competition for shared resources such as Fe and Mn [40].

Manganese homeostasis is intertwined with virulence across many species of bacteria [56, 59]. For example in *Yersinia pseudotuberculosis *, *mntH* is essential for bacterial survival within host cells [60]. In *N. gonorrhoeae,* the *mntABC* Mn transport system has been characterized as a virulence factor [61], and mutants of this transport system show defects in biofilm formation on human cervical cells [62]. Manganese concentrations have also been shown to affect expression of pili – a critical factor for bacterial cell adhesion to host cells [57]. Interestingly, increased concentrations of manganese represses expression of the T4SS encoded by the GGI [48], suggesting that *mntH* may have co-evolved with the GGI in order to calibrate manganese concentrations at a level that facilitates the expression and/or function of the GGI. Biochemical characterization of the different versions of MntH identified in this work (the versions with and without the 11 non-synonymous mutations) may help elucidate the role of MntH in manganese homeostasis, and even more interestingly, how this locus interacts with the GGI.

### Niche partitioning in *N. gonorrhoeae*

Previous work studying the genomics of pathogenic *Neisseria* has shown specific adaptation in cervical isolates relative to urethral isolates, indicating that *Neisseria* spp. are niche partitioned by body site [63]. Based on our observations that GGI+ and GGI− sub-populations appear to differ with respect to opportunities for HGT and potentially homeostasis of manganese, we hypothesize that these sub-populations have differential access to distinct niches within hosts. Given the involvement of the GGI and manganese transporters in biofilm formation [49, 57, 62], one possibility is that GGI+ and GGI− sub-populations differ with respect to their propensity to form biofilms, or the type of biofilms they form. *N. gonorrhoeae* forms biofilms *in vitro* on cervical and urethral epithelia [64], and biofilms have been observed forming on cervical biopsy specimens [65]. *N. gonorrhoeae* biofilms are natural reservoirs for HGT [66]. The increase in diversity we observe in GGI+ isolates may thus be facilitated by their propensity to form biofilms or perhaps to form biofilms with more diverse partners. The GGI is more prevalent among strains isolated from disseminated infections [5], which could be related to biofilm formation as biofilms provide protection from immune system clearance and enable growth of large, dense bacterial communities that may increase the risk of dissemination [67].

### GGI maintained at intermediate frequencies

The GGI is excised from the chromosome by site-specific recombination at *dif* sites by XerCD, and experimental data show that the GGI can be readily excised from the chromosome in a laboratory setting [3, 10]. In this study we provide evidence for the GGI’s mobility in natural populations: the diversity within the GGI – both in gene content (Fig. 7a) and gene sequence (Fig. 7c) – and the evidence of intra-locus recombination (Fig. 7b) suggest that not only is the GGI able to move in and out (gain of the GGI by GGI− strain or loss by GGI+ strain) but it may also be able to exchange GGI fragments without excision (Fig. 8c). And yet, the GGI is structured on the phylogeny (Fig. 1) and is not gained or lost at random. The GGI’s segregation at an intermediate frequency (61 % in this study) in the presence of abundant recombination and its capacity for mobilization suggest one or more mechanisms that keep the GGI from being lost or fixed in the population. Co-adaptation between MGEs and the host chromosome has been demonstrated many times in the experimental evolution of plasmids [68–73] where fitness costs associated with maintenance of the MGE are mitigated by compensatory mutations in the chromosome, the element or both. We find the *N. gonorrhoeae* chromosome has adapted with significant differences in *mntH*, and we hypothesize that co-adaption of the GGI with the *N. gonorrhoeae* core genome may limit mobility by increasing fitness costs associated with introduction of the GGI to a GGI− chromosomal background. Adaptation of *mntH* to function alongside the GGI may have allowed for the maintenance of the GGI in natural populations while also serving as a barrier to spread of the GGI to GGI− isolates.

Another possible contributing factor to the GGI’s segregation at intermediate frequencies is that the element is under some form of balancing selection, specifically negative frequency-dependent selection (NFDS), which occurs when the relative fitness of a variant decreases as its frequency in the population increases. Genes under NFDS are often found on genomic islands, as low linkage with the rest of the genome allows for rapid turnover [74, 75]. It is also common for genes that enable cheating (e.g. production of biofilm extracellular matrix (ECM) components such as ssDNA) to evolve under NFDS as cheaters evolve to escape the production cost of common goods [76]. As outlined above, our results suggest that GGI+ and GGI− sub-populations preferentially occupy slightly different niches, which would seem likely to preclude GGI− isolates from exploiting common goods – such as extracellular matrix – produced by GGI+ isolates. However, observations in this work and others show that GGI genes that are essential for the secretion of DNA (such as *atlA*) are not found at 100 % frequency in GGI+ isolates (Fig. 7a) [5, 37, 44, 47]. GGI+ isolates lacking the capability to secrete ssDNA could continue to inhabit biofilms alongside isolates with an intact GGI by cheating. This suggests that degradation of the GGI resulting in loss of T4SS functionality may be a signal of NFDS and that survival in *N. gonorrhoeae* biofilms may be partially dependent on other functions encoded by the GGI aside from secretion of ssDNA.

In summary, through analysis of natural populations of *N. gonorrhoeae* we have provided an analytical framework for studying MGEs in natural populations and demonstrated the importance of those elements in shaping bacterial populations. We have shown that the GGI has both shaped the gonococcal chromosome as well as allowing for sub-populations of *N. gonorrhoeae* to inhabit different niches. Applying the methods described in this work to larger, global datasets of *N. gonorrhoeae* genomes will help expand our knowledge of the diversity of the GGI and may reveal other ways in which the GGI functions to shape sub-populations of *N. gonorroheae*. This work emphasizes the importance of studying clinical isolates and expands on our knowledge of how bacteria adapt to diverse environments.

## Supplementary Data

Supplementary material 1

## References

[1] CDC (2021). CDC - Gonorrhea. https://www.cdc.gov/std/gonorrhea/stdfact-gonorrhea-detailed.htm.

[2] Koomey M (1998). Competence for natural transformation in *Neisseria gonorrhoeae*: a model system for studies of horizontal gene transfer. APMIS.

[3] Hamilton HL, Domínguez NM, Schwartz KJ, Hackett KT, Dillard JP (2005). *Neisseria gonorrhoeae* secretes chromosomal DNA via a novel type IV secretion system. Mol Microbiol.

[4] Salgado-Pabón W, Jain S, Turner N, van der Does C, Dillard JP (2007). A novel relaxase homologue is involved in chromosomal DNA processing for type IV secretion in *Neisseria gonorrhoeae*. Mol Microbiol.

[5] Dillard JP, Seifert HS (2001). A variable genetic island specific for *Neisseria gonorrhoeae* is involved in providing DNA for natural transformation and is found more often in disseminated infection isolates. Mol Microbiol.

[6] Pachulec E, Siewering K, Bender T, Heller E-M, Salgado-Pabon W (2014). Functional analysis of the gonococcal genetic island of *Neisseria gonorrhoeae*. PLoS One.

[7] Wu Z, Xu L, Tu Y, Chen R, Yu Y (2011). The relationship between the symptoms of female gonococcal infections and serum progesterone level and the genotypes of *Neisseria gonorrhoeae* multi-antigen sequence type (NG-MAST) in Wuhan, China. Eur J Clin Microbiol Infect Dis.

[8] Pachulec E, van der Does C (2010). Conjugative plasmids of *Neisseria gonorrhoeae*. PLoS One.

[9] Callaghan MM, Heilers J-H, van der Does C, Dillard JP (2017). Secretion of chromosomal DNA by the *Neisseria gonorrhoeae* type IV secretion system. Curr Top Microbiol Immunol.

[10] Domínguez NM, Hackett KT, Dillard JP (2011). XerCD-mediated site-specific recombination leads to loss of the 57-kilobase gonococcal genetic island. J Bacteriol.

[11] Menardo F, Loiseau C, Brites D, Coscolla M, Gygli SM (2018). Treemmer: a tool to reduce large phylogenetic datasets with minimal loss of diversity. BMC Bioinformatics.

[12] Andrews S. (2010). FastQC: A Quality Control tool for High Throughput Sequence Data.

[13] Bankevich A, Nurk S, Antipov D, Gurevich AA, Dvorkin M (2012). SPAdes: a new genome assembly algorithm and its applications to single-cell sequencing. J Comput Biol.

[14] Gurevich A, Saveliev V, Vyahhi N, Tesler G (2013). QUAST: quality assessment tool for genome assemblies. Bioinformatics.

[15] Seemann T (2014). Prokka: rapid prokaryotic genome annotation. Bioinformatics.

[16] Page AJ, Cummins CA, Hunt M, Wong VK, Reuter S (2015). Roary: rapid large-scale prokaryote pan genome analysis. Bioinformatics.

[17] Stamatakis A (2014). RAxML version 8: a tool for phylogenetic analysis and post-analysis of large phylogenies. Bioinformatics.

[18] Méric G, Yahara K, Mageiros L, Pascoe B, Maiden MCJ (2014). A reference pan-genome approach to comparative bacterial genomics: identification of novel epidemiological markers in pathogenic *Campylobacter*. PLoS One.

[19] Page AJ, Taylor B, Delaney AJ, Soares J, Seemann T (2016). *SNP-sites*: rapid efficient extraction of SNPs from multi-FASTA alignments. Microb Genom.

[20] Huson DH, Bryant D (2006). Application of phylogenetic networks in evolutionary studies. Mol Biol Evol.

[21] Madden T (2003). The BLAST Sequence Analysis ToolThe NCBI Handbook [Internet]. National Center for Biotechnology Information (US). https://www.ncbi.nlm.nih.gov/books/NBK21097.

[22] Guy L, Kultima JR, Andersson SGE (2010). genoPlotR: comparative gene and genome visualization in R. Bioinformatics.

[23] Croucher NJ, Page AJ, Delaney AJ, Keane JA (2015). Rapid phylogenetic analysis of large samples of recombinant bacterial whole genome sequences using Gubbins. Nucleic Acids Res.

[24] Hadfield J, Croucher NJ, Goater RJ, Abudahab K, Aanensen DM (2018). Phandango: an interactive viewer for bacterial population genomics. Bioinformatics.

[25] Yu G, Smith DK, Zhu H, Guan Y, Lam T-Y (2017). Ggtree: an R package for visualization and annotation of phylogenetic trees with their covariates and other associated data. Methods Ecol Evol.

[26] Brynildsrud O, Bohlin J, Scheffer L, Eldholm V (2016). Rapid scoring of genes in microbial pan-genome-wide association studies with Scoary. Genome Biol.

[27] Sagulenko P, Puller V, Neher RA (2018). Treetime: maximum-likelihood phylodynamic analysis. Virus Evol.

[28] Reis-Cunha JL, Bartholomeu DC, Manson AL, Earl AM, Cerqueira GC (2019). ProphET, prophage estimation tool: a stand-alone prophage sequence prediction tool with self-updating reference database. PLoS One.

[29] Ondov BD, Treangen TJ, Melsted P, Mallonee AB, Bergman NH (2016). Mash: fast genome and metagenome distance estimation using MinHash. Genome Biol.

[30] Johansson MHK, Bortolaia V, Tansirichaiya S, Aarestrup FM, Roberts AP (2021). Detection of mobile genetic elements associated with antibiotic resistance in Salmonella enterica using a newly developed web tool: MobileElementFinder. J Antimicrob Chemother.

[31] Löytynoja A (2014). Phylogeny-aware alignment with PRANK. Methods Mol Biol.

[32] De Mita S, Siol M (2012). EggLib: processing, analysis and simulation tools for population genetics and genomics. BMC Genet.

[33] Jombart T (2008). adegenet: a R package for the multivariate analysis of genetic markers. Bioinformatics.

[34] Paradis E, Claude J, Strimmer K (2004). APE: Analyses of Phylogenetics and Evolution in R language. Bioinformatics.

[35] Mortimer TD, Pathela P, Crawley A, Rakeman JL, Lin Y (2021). The distribution and spread of susceptible and resistant *Neisseria gonorrhoeae* across demographic groups in a major metropolitan center. Clin Infect Dis.

[36] Golparian D, Harris SR, Sánchez-Busó L, Hoffmann S, Shafer WM (2020). Genomic evolution of *Neisseria gonorrhoeae* since the preantibiotic era (1928-2013): antimicrobial use/misuse selects for resistance and drives evolution. BMC Genomics.

[37] Shaskolskiy B, Kravtsov D, Kandinov I, Gorshkova S, Kubanov A (2022). Comparative whole-genome analysis of *Neisseria gonorrhoeae* isolates revealed changes in the gonococcal genetic island and specific genes as a link to antimicrobial resistance. Front Cell Infect Microbiol.

[38] Hamilton HL, Dillard JP (2006). Natural transformation of *Neisseria gonorrhoeae*: from DNA donation to homologous recombination. Mol Microbiol.

[39] Hohle TH, O’Brian MR (2009). The mntH gene encodes the major Mn(2+) transporter in *Bradyrhizobium japonicum* and is regulated by manganese via the Fur protein. Mol Microbiol.

[40] Kehres DG, Zaharik ML, Finlay BB, Maguire ME (2000). The NRAMP proteins of *Salmonella typhimurium* and *Escherichia coli* are selective manganese transporters involved in the response to reactive oxygen. Mol Microbiol.

[41] Tseng H-J, Srikhanta Y, McEwan AG, Jennings MP (2001). Accumulation of manganese in *Neisseria gonorrhoeae* correlates with resistance to oxidative killing by superoxide anion and is independent of superoxide dismutase activity. Mol Microbiol.

[42] Mortimer TD, Weber AM, Pepperell CS (2018). Signatures of selection at drug resistance loci in *Mycobacterium tuberculosis*. mSystems.

[43] Piekarowicz A, Kłyz A, Majchrzak M, Adamczyk-Popławska M, Maugel TK (2007). Characterization of the dsDNA prophage sequences in the genome of *Neisseria gonorrhoeae* and visualization of productive bacteriophage. BMC Microbiol.

[44] Woodhams KL, Benet ZL, Blonsky SE, Hackett KT, Dillard JP (2012). Prevalence and detailed mapping of the gonococcal genetic island in *Neisseria meningitidis*. J Bacteriol.

[45] Hamilton HL, Schwartz KJ, Dillard JP (2001). Insertion-duplication mutagenesis of neisseria: use in characterization of DNA transfer genes in the gonococcal genetic island. J Bacteriol.

[46] Kohler PL, Hamilton HL, Cloud-Hansen K, Dillard JP (2007). AtlA functions as a peptidoglycan lytic transglycosylase in the *Neisseria gonorrhoeae* type IV secretion system. J Bacteriol.

[47] Kohler PL, Chan YA, Hackett KT, Turner N, Hamilton HL (2013). Mating pair formation homologue TraG is a variable membrane protein essential for contact-independent type IV secretion of chromosomal DNA by *Neisseria gonorrhoeae*. J Bacteriol.

[48] Callaghan MM, Klimowicz AK, Shockey AC, Kane J, Pepperell CS (2021). Transcriptional and translational responsiveness of the *Neisseria gonorrhoeae* type IV secretion system to conditions of host infections. Infect Immun.

[49] Zweig M, Schork S, Koerdt A, Siewering K, Sternberg C (2014). Secreted single-stranded DNA is involved in the initial phase of biofilm formation by *Neisseria gonorrhoeae*. Environ Microbiol.

[50] Bobay L-M, Ochman H (2018). Factors driving effective population size and pan-genome evolution in bacteria. BMC Evol Biol.

[51] Moran NA (2002). Microbial minimalism: genome reduction in bacterial pathogens. Cell.

[52] Murray GGR, Charlesworth J, Miller EL, Casey MJ, Lloyd CT (2021). Genome reduction is associated with bacterial pathogenicity across different scales of temporal and ecological divergence. Mol Biol Evol.

[53] Newton ILG, Bordenstein SR (2011). Correlations between bacterial ecology and mobile DNA. Curr Microbiol.

[54] Weinert LA, Welch JJ (2017). Why might bacterial pathogens have small genomes?. Trends Ecol Evol.

[55] Bohr LL, Youngblom MA, Eldholm V, Pepperell CS (2021). Genome reorganization during emergence of host-associated *Mycobacterium abscessus*. Microb Genom.

[56] Bosma EF, Rau MH, van Gijtenbeek LA, Siedler S (2021). Regulation and distinct physiological roles of manganese in bacteria. FEMS Microbiol Rev.

[57] Wu H-J, Seib KL, Srikhanta YN, Edwards J, Kidd SP (2010). Manganese regulation of virulence factors and oxidative stress resistance in *Neisseria gonorrhoeae*. J Proteomics.

[58] Forbes JR, Gros P (2001). Divalent-metal transport by NRAMP proteins at the interface of host-pathogen interactions. Trends Microbiol.

[59] Perry RD, Craig SK, Abney J, Bobrov AG, Kirillina O (2012). Manganese transporters Yfe and MntH are Fur-regulated and important for the virulence of *Yersinia pestis*. Microbiology.

[60] Champion OL, Karlyshev A, Cooper IAM, Ford DC, Wren BW (2011). *Yersinia pseudotuberculosis* mntH functions in intracellular manganese accumulation, which is essential for virulence and survival in cells expressing functional Nramp1. Microbiology.

[61] Quillin SJ, Seifert HS (2018). *Neisseria gonorrhoeae* host adaptation and pathogenesis. Nat Rev Microbiol.

[62] Falsetta ML, Steichen CT, McEwan AG, Cho C, Ketterer M (2011). The composition and metabolic phenotype of *Neisseria gonorrhoeae* biofilms. Front Microbiol.

[63] Ma KC, Mortimer TD, Hicks AL, Wheeler NE, Sánchez-Busó L (2020). Adaptation to the cervical environment is associated with increased antibiotic susceptibility in *Neisseria gonorrhoeae*. Nat Commun.

[64] Greiner LL, Edwards JL, Shao J, Rabinak C, Entz D (2005). Biofilm formation by *Neisseria gonorrhoeae*. Infect Immun.

[65] Steichen CT, Shao JQ, Ketterer MR, Apicella MA (2008). Gonococcal cervicitis: a role for biofilm in pathogenesis. J Infect Dis.

[66] Kouzel N, Oldewurtel ER, Maier B (2015). Gene transfer efficiency in gonococcal biofilms: role of biofilm age, architecture, and pilin antigenic variation. J Bacteriol.

[67] Vestby LK, Grønseth T, Simm R, Nesse LL (2020). Bacterial biofilm and its role in the pathogenesis of disease. Antibiotics.

[68] Bouma JE, Lenski RE (1988). Evolution of a bacteria/plasmid association. Nature.

[69] De Gelder L, Williams JJ, Ponciano JM, Sota M, Top EM (2008). Adaptive plasmid evolution results in host-range expansion of a broad-host-range plasmid. Genetics.

[70] Zwanzig M, Harrison E, Brockhurst MA, Hall JPJ, Berendonk TU (2019). Mobile compensatory mutations promote plasmid survival. mSystems.

[71] Stalder T, Rogers LM, Renfrow C, Yano H, Smith Z (2017). Emerging patterns of plasmid-host coevolution that stabilize antibiotic resistance. Sci Rep.

[72] Dahlberg C, Chao L (2003). Amelioration of the cost of conjugative plasmid carriage in *Eschericha coli* K12. Genetics.

[73] San Millan A, Toll-Riera M, Qi Q, MacLean RC (2015). Interactions between horizontally acquired genes create a fitness cost in *Pseudomonas aeruginosa*. Nat Commun.

[74] Jones JM, Grinberg I, Eldar A, Grossman AD (2021). A mobile genetic element increases bacterial host fitness by manipulating development. Elife.

[75] Makarova KS, Wolf YI, Snir S, Koonin EV (2011). Defense islands in bacterial and archaeal genomes and prediction of novel defense systems. J Bacteriol.

[76] Cordero OX, Polz MF (2014). Explaining microbial genomic diversity in light of evolutionary ecology. Nat Rev Microbiol.

