# Supplementary material for: The Gonococcal Genetic Island defines distinct sub-populations of Neisseria gonorrhoeae
Source: Microb Genom. 2023 May 22;9(5):mgen000985. doi: 10.1099/mgen.0.000985 (PMC10272882; doi:10.1099/mgen.0.000985)
Supplement: Supplementary material 1 [file mgen-9-985-s001.pdf]

The Gonococcal Genetic Island defines distinct sub-populations of *Neisseria gonorrhoeae*

Supplement

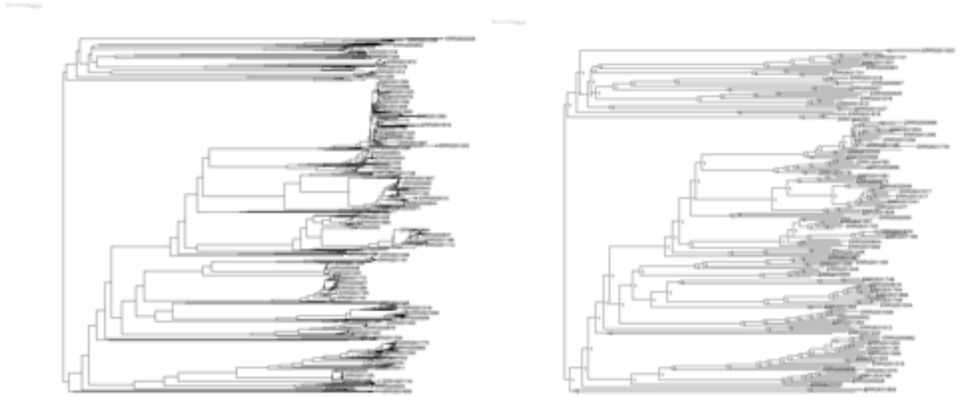

Figure S1: Phylogenetic tree of dataset before (left) and after (right) subsampling. Dataset started with 860 isolates and was subsampled while maintaining maximum diversity to 200 isolates using Treemmer.

Table S1: 201 isolates used in this study listed by NCBI SRA accession and GGI status. A raw text version of this table is also available here: https://github.com/myoungblom/Evolution_of_the_GGI.

SRA	GGI	SRA cont.	GGI cont.
ERR1204778	neg	ERR2631831	pos
ERR1204783	neg	ERR2631843	pos
ERR1204788	neg	ERR2631912	pos
ERR1837107	neg	ERR3200920	pos
ERR1837111	neg	ERR3200927	pos
ERR1837123	neg	ERR3201129	pos
ERR2631728	neg	ERR3201195	pos
ERR2631731	neg	ERR3201216	pos
ERR2631746	neg	ERR3201231	pos
ERR2631748	neg	ERR3201317	pos
ERR2631769	neg	ERR2631725	pos
ERR2631778	neg	ERR2631734	pos
ERR2631794	neg	ERR2631796	pos
ERR2631807	neg	ERR2631833	pos

ERR2631829	neg	ERR2631838	pos
ERR2631855	neg	ERR2631868	pos
ERR2631859	neg	ERR2631870	pos
ERR2631880	neg	ERR2631887	pos
ERR2631895	neg	ERR2631891	pos
ERR2631901	neg	ERR2631907	pos
ERR2631903	neg	ERR2631929	pos
ERR2631904	neg	ERR2631930	pos
ERR2631915	neg	ERR2631938	pos
ERR2631917	neg	ERR2631942	pos
ERR2631918	neg	ERR2631975	pos
ERR2631925	neg	ERR2631980	pos
ERR2631927	neg	ERR2631984	pos
ERR2631981	neg	ERR2632006	pos
ERR2631987	neg	ERR2632037	pos
ERR2631996	neg	ERR3200821	pos
ERR2631998	neg	ERR3200831	pos
ERR2631999	neg	ERR3200866	pos
ERR2632000	neg	ERR3200873	pos
ERR2632008	neg	ERR3200894	pos
ERR2632009	neg	ERR3200896	pos
ERR2632020	neg	ERR3200901	pos
ERR2632040	neg	ERR3200904	pos
ERR2632042	neg	ERR3200906	pos
ERR3200818	neg	ERR3200928	pos
ERR3200855	neg	ERR3200931	pos
ERR3200864	neg	ERR3200982	pos
ERR3200868	neg	ERR3201011	pos
ERR3200892	neg	ERR3201024	pos
ERR3200918	neg	ERR3201035	pos
ERR3200929	neg	ERR3201050	pos
ERR3200944	neg	ERR3201052	pos
ERR3200946	neg	ERR3201066	pos
ERR3200955	neg	ERR3201069	pos
ERR3200968	neg	ERR3201078	pos
ERR3200973	neg	ERR3201088	pos
ERR3201004	neg	ERR3201137	pos
ERR3201017	neg	ERR3201150	pos
ERR3201034	neg	ERR3201166	pos

ERR3201041	neg	ERR3201169	pos
ERR3201046	neg	ERR3201177	pos
ERR3201051	neg	ERR3201179	pos
ERR3201070	neg	ERR3201184	pos
ERR3201077	neg	ERR3201186	pos
ERR3201081	neg	ERR3201219	pos
ERR3201093	neg	ERR3201235	pos
ERR3201094	neg	ERR3201242	pos
ERR3201116	neg	ERR3201273	pos
ERR3201128	neg	ERR3201286	pos
ERR3201130	neg	ERR3201291	pos
ERR3201163	neg	ERR3201349	pos
ERR3201209	neg	ERR3201370	pos
ERR3201217	neg	NCCP11945	pos
ERR3201258	neg	ERR1204792	pos
ERR3201260	neg	ERR1204796	pos
ERR3201272	neg	ERR2631763	pos
ERR3201281	neg	ERR2631768	pos
ERR3201282	neg	ERR2631813	pos
ERR3201283	neg	ERR2631819	pos
ERR3201287	neg	ERR2631834	pos
ERR3201290	neg	ERR2631849	pos
ERR3201296	neg	ERR2631913	pos
ERR3201337	neg	ERR2631965	pos
ERR3201361	neg	ERR2632021	pos
ERR3200996	pos	ERR3200814	pos
ERR2631876	pos	ERR3200846	pos
ERR2632022	pos	ERR3200854	pos
ERR3200816	pos	ERR3200885	pos
ERR3200852	pos	ERR3200893	pos
ERR3200899	pos	ERR3200907	pos
ERR3200930	pos	ERR3201018	pos
ERR3200981	pos	ERR3201030	pos
ERR3201038	pos	ERR3201096	pos
ERR3201121	pos	ERR3201234	pos
ERR3201151	pos	ERR3201246	pos
ERR3201201	pos	ERR3201301	pos
ERR3201227	pos	ERR3201308	pos
ERR3201269	pos	ERR3201346	pos

ERR3201322	pos	ERR2631964	pos
ERR1837113	pos	ERR3200876	pos
ERR2632028	pos	ERR3200936	pos
ERR3200913	pos	ERR3200952	pos
ERR3200919	pos	ERR3200960	pos
ERR3201303	pos	ERR3201008	pos
ERR3201315	pos	ERR3201098	pos
ERR1837114	pos	ERR3201284	pos
ERR1837132	pos		

Table S2: Core genome analysis and global diversity estimates. Average gene number per isolate and gene content distribution within the core (found in ≥ 99% isolates), soft core (95% ≤ frequency < 99%), shell (15% ≤ frequency < 95%) and cloud genomes (frequency < 15%), as calculated by Roary. Diversity statistics for core genome alignments including theta, pi and Tajima's D calculated using Egglib.

Sample	N	Total Genes	Average Gene Number	Core Genes	Soft Core Genes	Shell Genes	Cloud Genes	θ	π	Tajima's D
All	201	3205	2078	1742	83	369	1011	1.65e-2	7.45e-3	-1.77
GGI+	123	3078	2108	1788	87	327	885	1.49e-2	7.78e-3	-1.61
GGI-	78	2677	2031	1731	119	288	539	8.67e-3	6.78e-3	-0.760
GGI	123	61	60	53	3	4	1	3.73e-2	1.56e-2	-1.96

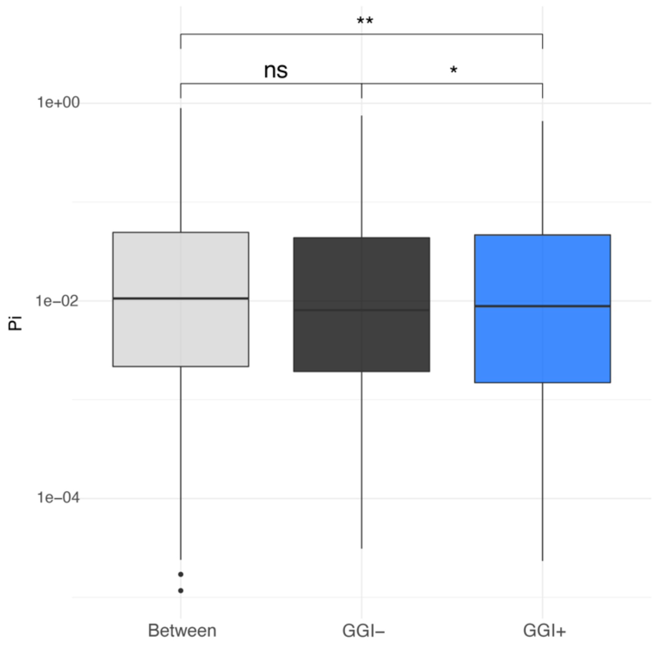

Figure S2: Nucleotide diversity (pi) calculated using egglib for accessory gene clusters as defined by Roary, both within and between GGI+ and GGI- groups. Mann-Whitney U test with Benjamini-Hochberg correction indicates that diversity of accessory genes within GGI+ isolates is slightly lower than that of GGI- isolates, as well as being lower than diversity compared across groups. P-value legend: *: <=0.05, **: <=0.01

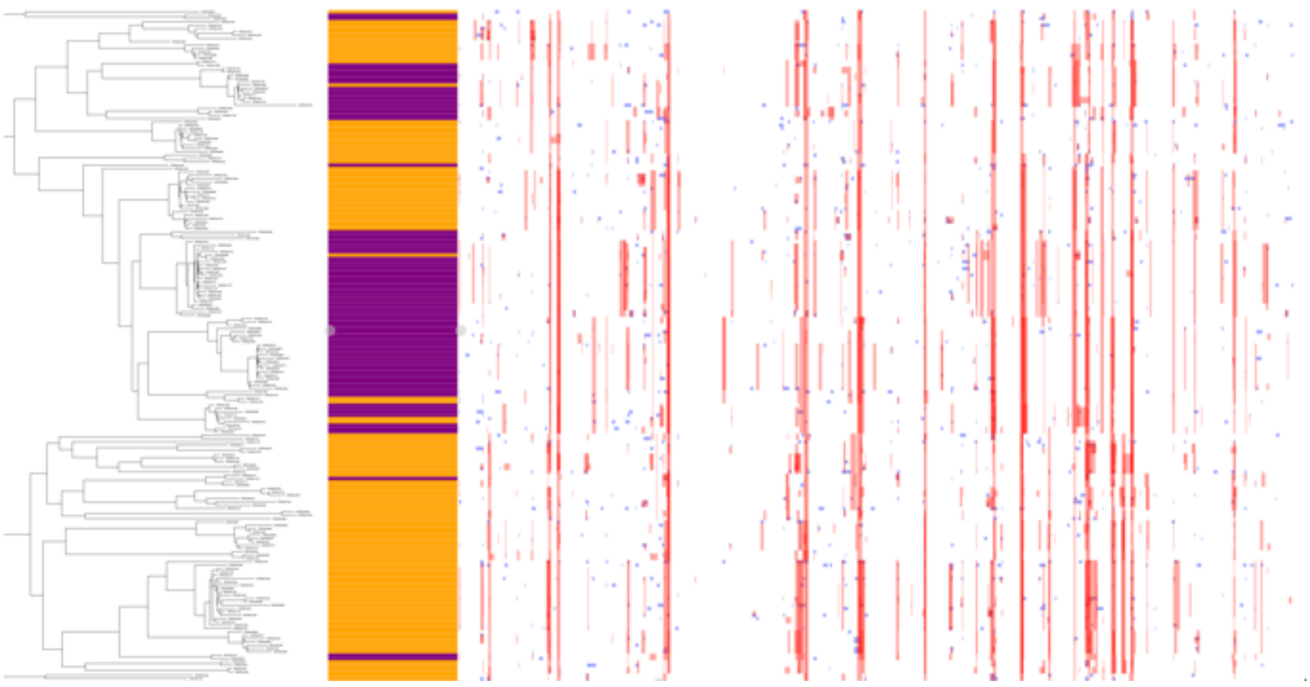

Figure S3: Recombinant fragments in the core genome of *N. gonorrhoeae* identified by Gubbins and plotted next to the core genome phylogeny in Phandango. Colors adjacent to tree tips represent GGI status: orange are GGI+ and purple are GGI- isolates. Recombinant fragments shared between isolates within the dataset are shown in red, while those which are determined to have originated outside of the dataset are in blue. 81% of the total core genome alignment is predicted to be in a recombinant fragment in at least one isolate in this dataset.

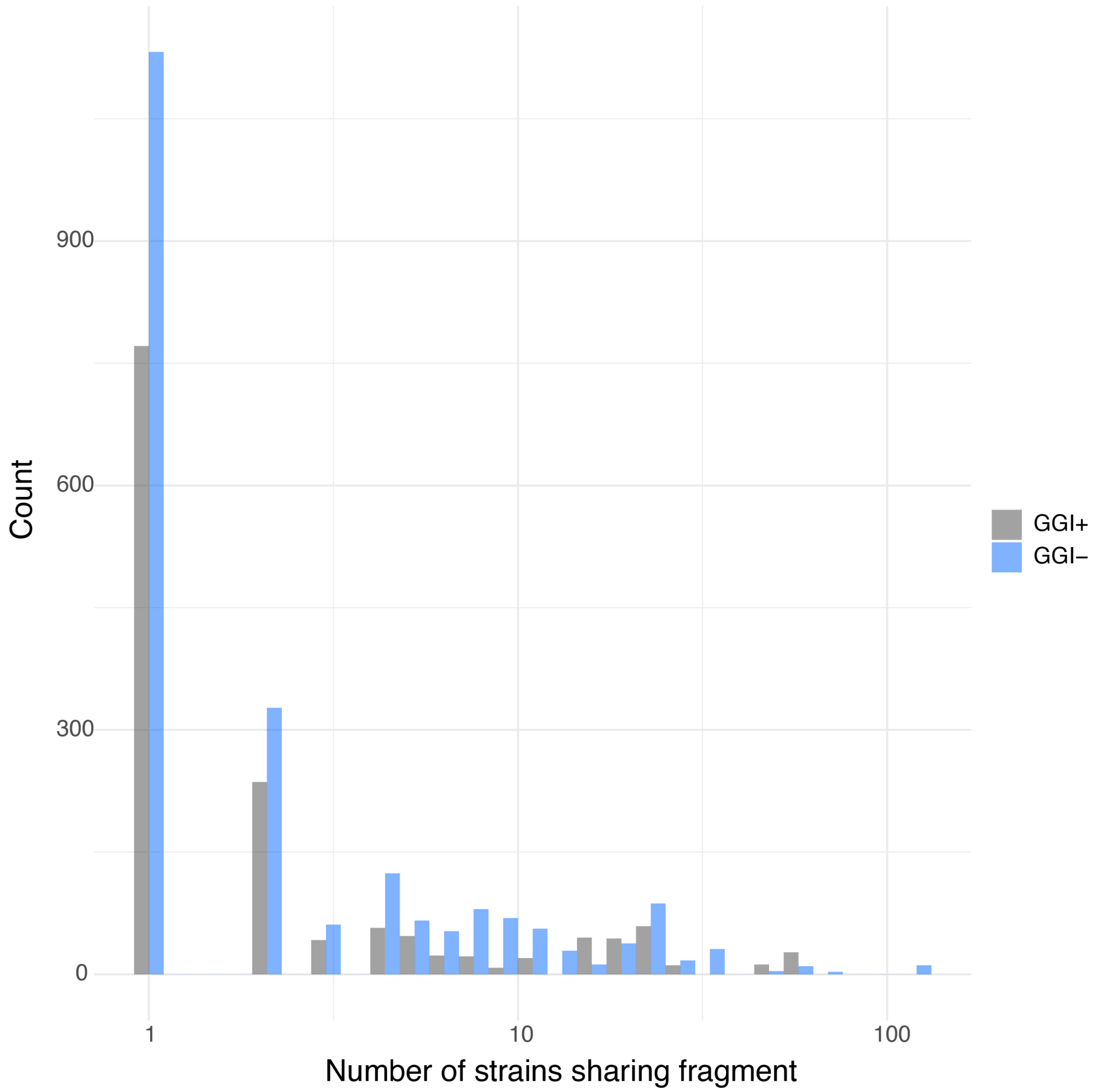

Figure S4: Histogram showing the number of taxa within each group sharing a particular recombinant fragment. Distributions across GGI+ and GGI- isolates are similar indicating that isolates from a particular group are not more likely to share the same recombinant fragments.

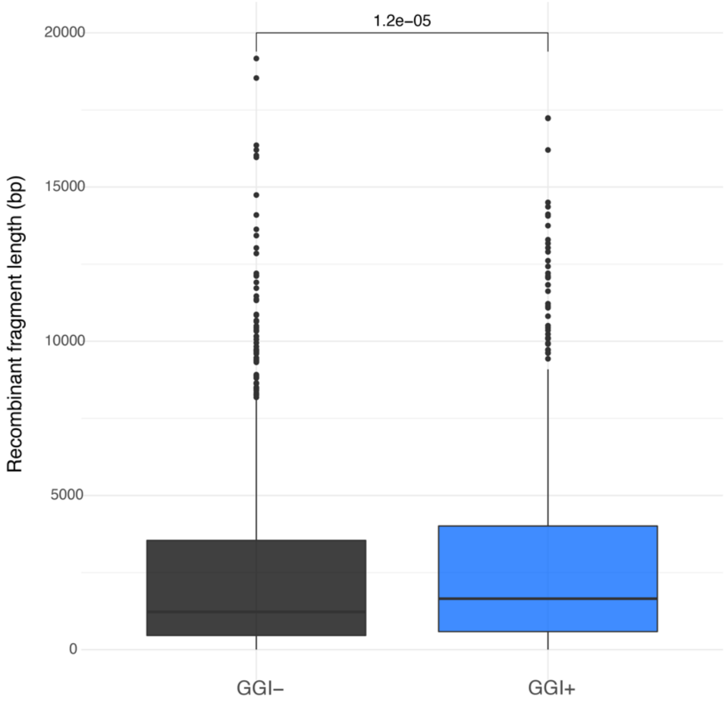

Figure S5: Recombinant fragment lengths as calculated by Gubbins are significantly longer in GGI+ isolates than in GGI- isolates (Mann-Whitney U test p-value=1.2e-05). Median values for recombinant fragment length are 1225 and 1653 for GGI- and GGI+ isolates, respectively.

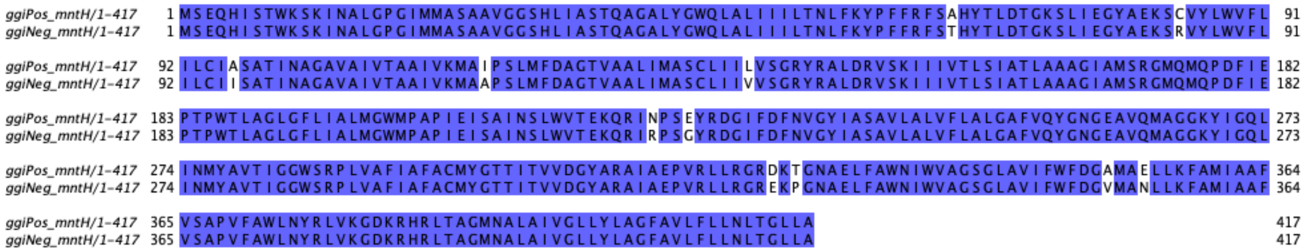

Figure S6: Amino acid alignment created using Clustal Omega (https://www.ebi.ac.uk/Tools/msa/clustalo/) of *mntH* which contained the 12 most significant F_{ST} outliers. Sequences from representative GGI+ and GGI- isolates show the resulting 11 non-synonymous changes. Visualization performed with Jalview and amino acid variants are colored in white.

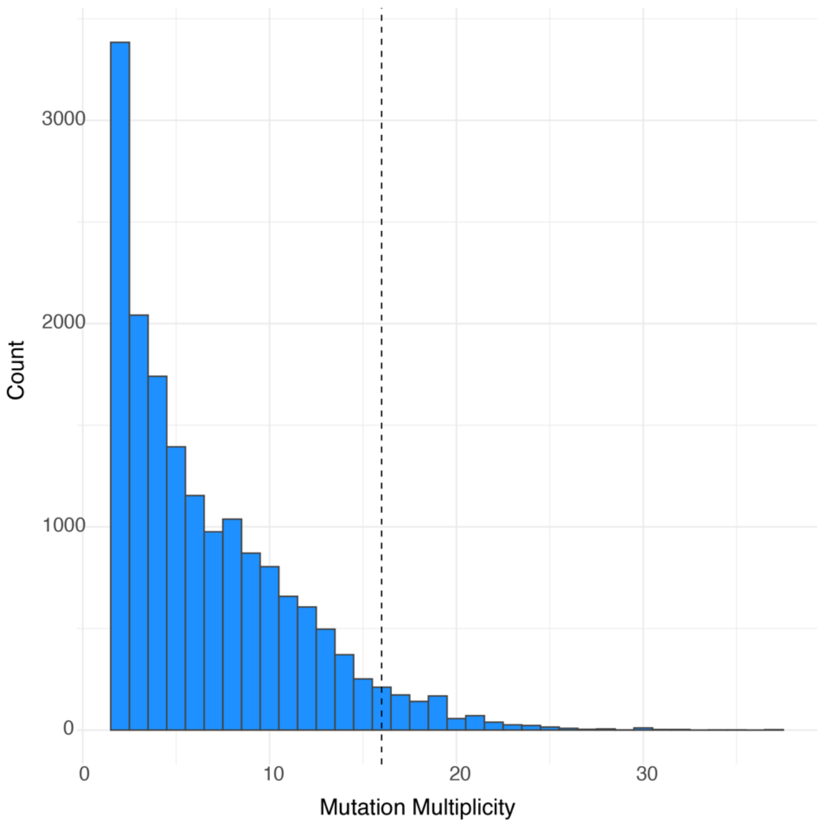

Figure S7: Distribution of homoplasy multiplicity with the 95th percentile being 16 and above (marked by black dotted line). Homoplastic mutations from core genome alignment identified using TreeTime.

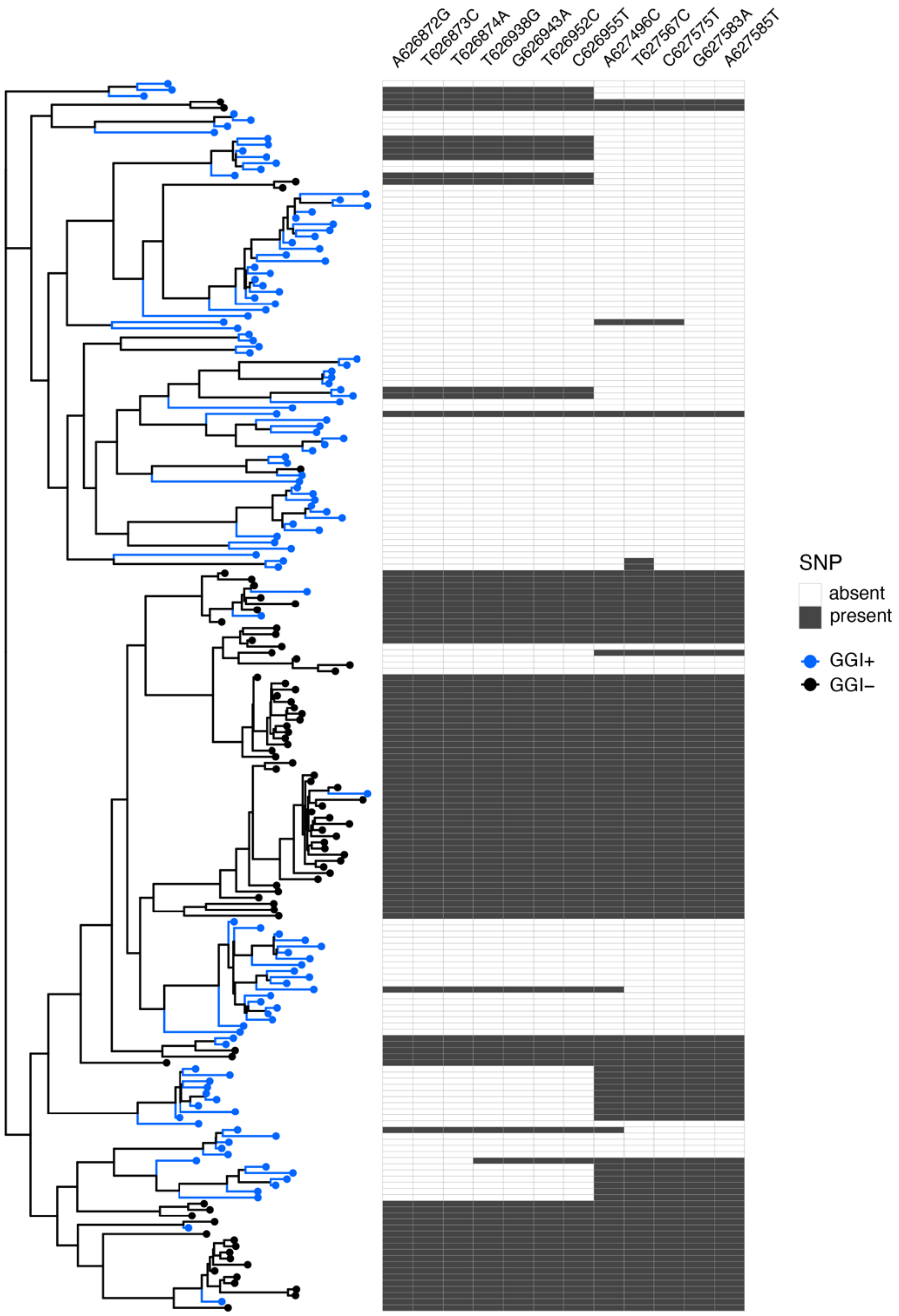

Figure S8: wcFst outliers in *mntH* show possible lineage effects when plotted next to the core genome phylogeny. Each column is a wcFst outlier, with column label showing reference (dominant) allele, core genome position and alternate allele. Each row corresponds to an isolate on the tree, tips colored by GGI presence/absence, and matrix position for each variant colored white to indicate the reference (dominant) allele, or grey for the alternate allele.

Table S3: Accessory genes identified as significantly associated with the GGI presence or absence by Scoary. Two phage genes of interest are highlighted in green. Annotations found by BLAST search of protein sequences and pulling annotations from the top results.

Annotation	GGI+ Frequency	GGI- Frequency	Naive_p	Bonferroni_p
phage tail protein	0.951219512	0.371794872	8.67E-20	8.23E-17
phage recombination protein ninB	0.829268293	0.320512821	3.36E-13	3.19E-10
hypothetical protein	0.845528455	0.243589744	8.66E-18	8.22E-15
hemagglutinin repeat containing protein	0.804878049	0.243589744	2.25E-15	2.14E-12
hypothetical protein	0.195121951	0.756410256	2.25E-15	2.14E-12
hypothetical protein	0.821138211	0.294871795	6.37E-14	6.05E-11
hypothetical protein	0.650406504	0.141025641	3.60E-13	3.42E-10
hypothetical protein	0.512195122	0.064102564	4.41E-12	4.18E-09
hypothetical protein	0.512195122	0.076923077	3.91E-11	3.71E-08
hypothetical protein	0.918699187	0.487179487	7.31E-12	6.94E-09
